# Objects with three orthogonal symmetry planes: Oblique driving forces and Stokes flow motion

**DOI:** 10.1371/journal.pone.0352508

**Published:** 2026-07-06

**Authors:** Robert J. Deissler, Robert Brown

**Affiliations:** Department of Physics, Case Western Reserve University, Cleveland, Ohio, United States of America; University at Buffalo, UNITED STATES OF AMERICA

## Abstract

Stokes flow studies are fundamental to advancing medical and industrial technologies, particularly in areas such as drug targeting, cell studies, the optimization of drug carrier vehicles, high viscosity flows, and magnetic particle imaging. While previous research has focused on the motion of obliquely falling cylindrical rods and magnetic particle chains, a broader analytical framework is required to understand more complex particle-fluid migrations. In this paper, we first generalize the two-dimensional motion of an obliquely falling rod in a gravitational field to the three-dimensional motion of an object possessing three mutually perpendicular planes of symmetry falling through a viscous fluid in the Stokes limit. We derive a general formula for the three components of velocity—including both downward and sideways components—for objects of arbitrary orientation and uniform density. These analytical solutions are defined in terms of the object’s orientation, specified via Euler angles, and the velocity of the object falling along each of its three principal axes, or the drag coefficient along each of those axes. We give a variety of examples of objects that satisfy this general formula. In addition, we apply the formula to a cuboid for which those velocity components along each of its principal axes have been measured experimentally by other researchers, thus giving both the downward and sideways components for arbitrary orientation. We then analyze the motion in a gradient magnetic field of elongated magnetic particles, such as nanorods and nanoellipsoids, for which the induced magnetic moment is along the long axis of the particle. We discuss the similarities and differences with the gravitational case. By providing a unified framework for predicting the trajectories of these symmetric bodies, this work enhances the understanding of the motion of inertial and magnetic particles under the influence of gravitational and gradient magnetic fields, respectively.

## 1. Introduction

The study of low Reynolds number flows, such as the motion of particles of micron and smaller sizes suspended in a fluid under the influence of force-fields, is of increasing importance in many applications. Examples of such flows are the motion of magnetic particle chains in a gradient magnetic field [1,2], the settling of aerosol particles in a gravitational field [3], the use of magnetic nanorods and nanoellipsoids in applications such as magnetic drug delivery and hyperthermia [4–8], propulsion of organisms and robots in granular media [9], non-Newtonian fluid flow in metallurgy [10], rheological properties of hollow magnetite chains [11], nanofluid flow through a porous diseased bifurcated artery [12], sedimentation of Brownian chains [13], Stokes flow of protein crystals toward a magnet source [14], as well as vertical and lateral drag on cellular membranes [15]. Recent reviews [16–19] also draw attention to the use of magnetic particles, particularly in the manipulation of cells in microfluidic devices, including their capture, and in targeted drug delivery.

### 1.1. Previous studies on drag effects

Yang et al. [1] presented experimental work measuring the drag coefficients of micron-sized magnetic beads and chains of such beads. These measurements were compared to various analytical predictions – ellipsoid, cylinder, and bead models – and also to the HYDRO++ numerical program [20–22]. This program was most successful in the description of the translational drag coefficients for a chain moving in a direction parallel to its length and for a chain moving in a direction perpendicular to its length. There was good agreement over a range of bead numbers making up a chain.

From the work on obliquely falling rods in a gravitational field in a viscous fluid [23–25], a richer motional picture was expected from the findings of *lateral motion* for falling rods [23–25]. In [2] we studied the motion of magnetic particle chains in a gradient magnetic field for which a chain makes an *arbitrary angle* with the magnetic force vector. Indeed, away from the parallel and perpendicular limits, we found that there was a sideways component of velocity. We derived analytical expressions for the components of the normalized velocity both parallel and perpendicular to the magnetic force in terms of the normalized velocity of a chain that is parallel to the magnetic force, the normalized velocity of a chain that is perpendicular to the magnetic force, and the angle that the chain makes with the magnetic force. In addition to the magnetic chain cases, we also studied the case of an obliquely falling rod in a viscous fluid in a gravitational field [2]. We derived more general expressions than those found in the literature [23–25], which consider only the deflection angle, and then only for the special case of a long thin rod, where the velocity of a vertically falling rod is twice that of a horizontally falling rod. In [2] we derived equations for the horizontal and vertical components of the velocity for a circular cylindrical rod with an *arbitrary aspect ratio* that makes an arbitrary angle with the vertical in terms of the velocity of a vertically falling rod, the velocity of a horizontally falling rod, and the angle that the rod makes to the vertical. In addition, we derived equations for the deflection angle for an arbitrary aspect ratio. We confirmed that as the rod is drawn in the direction of the gravitational force, the rod is also deflected sideways because of the interaction of the rod with the fluid, causing a component of velocity perpendicular to the gravitational force vector. This sideways motion corresponds to the presence of off-diagonal elements in the mobility matrix.

### 1.2. Present studies on drag effects

In view of the growing role of microparticles and nanoparticles under force-field influence in various applications, it continues to be important to understand more generally the flow dynamics. In this paper, we extend the analysis of [2] to three dimensions by deriving expressions for the components of velocity for an object with three mutually perpendicular planes of symmetry falling (due to gravity) through a fluid in the Stokes flow limit. Euler angles are used to specify the orientation of the object in 3D space. Expressions for its three components of velocity are derived in terms of the velocities of the object falling parallel to each of its three mutually perpendicular principal axes and in terms of the Euler angles specifying its orientation. Also, expressions are derived for the deflection angles. After a general theoretical discussion, examples of objects with three mutually perpendicular planes of symmetry are presented, such as cuboids, ellipsoids, and cylinders with more general cross sections. In addition, special cases are considered where the drag coefficients along two or three principal axes of the object are equal. The benefits of this general study include the challenge that would otherwise exist in understanding and quantifying motion for the more complex objects. The analogous analysis is also applied to a gradient magnetic field force on magnetic objects, with sideways motion for nonspherical cases. The equations derived in [2] for magnetic particles apply specifically to chains of spherical particles. In this paper we derive equations that describe the motion in a gradient magnetic field of magnetic particles of various shapes, including nanorods and nanoellipsoids, for which the induced magnetic moment is along the long axis of the particle.

## 2. Theory

Consider an object in a gravitational field falling through a viscous fluid for Reynolds number Re=ρUℓ/η→0 (in practice, Re≪1), where ρ is the density of the fluid, *U* is the speed of the object relative to the fluid, ℓ is a characteristic length of the object, and η is the dynamic viscosity of the fluid. This type of flow, referred to as Stokes flow or creeping flow, assumes that viscous forces dominate and inertial forces can be neglected. This approximation is seen to be excellent for all the examples discussed in the Introduction. Further, assume that the object has uniform density and has three mutually perpendicular planes of symmetry. In this case, the translational motion and the rotational motion are completely decoupled from one another, and the object maintains its orientation as it falls through the fluid, assuming that no torque is applied to the object [23].

In the Stokes flow limit, the velocity **U** of an object falling through a viscous fluid is related to the external force **F** acting on the object through the mobility matrix μ as [22,26]


$$\begin{array}{c} U=\mu \cdot F, \end{array}$$ (1)

where the force **F** is equal to the effective weight $W_{eff}$ of the object, that is, the gravitational force minus the buoyant force. Since we are in the Stokes flow limit Re→0, the time for the object to reach its terminal velocity after it is released approaches zero. Therefore, time does not appear in any of the equations, and we refer to **U** simply as the velocity. In general, the mobility matrix is a 6 x 6 matrix containing both translational and rotational components. Because, as noted previously, the translational motion completely decouples from the rotational motion for an object with three mutually perpendicular planes of symmetry and since only the translational motion is relevant here, we need only consider the translational components of the mobility matrix. Examples of an object with three mutually perpendicular planes of symmetry are a cuboid, an ellipsoid, and a rod with a cross section that has two mutually orthogonal lines of symmetry, such as elliptical, circular, rectangular, and hexagonal cross sections. From symmetry, if the object is oriented such that it is falling parallel to any of its three principal axes, the object will fall straight down, assuming it is far from boundaries and of uniform density [23]. This implies that **U** and **F** are parallel to one another, and therefore μ is diagonal. For any other orientation of the object μ will have off-diagonal elements and the object will have sideways components of its velocity.

The inverse of the mobility matrix is defined as the friction matrix $\zeta =\mu^{-1}$, which relates the velocity of the chain to the net force of the chain on the fluid as [22,26,27]


$$\begin{array}{c} F=\zeta \cdot U. \end{array}$$ (2)

**Fig 1:**
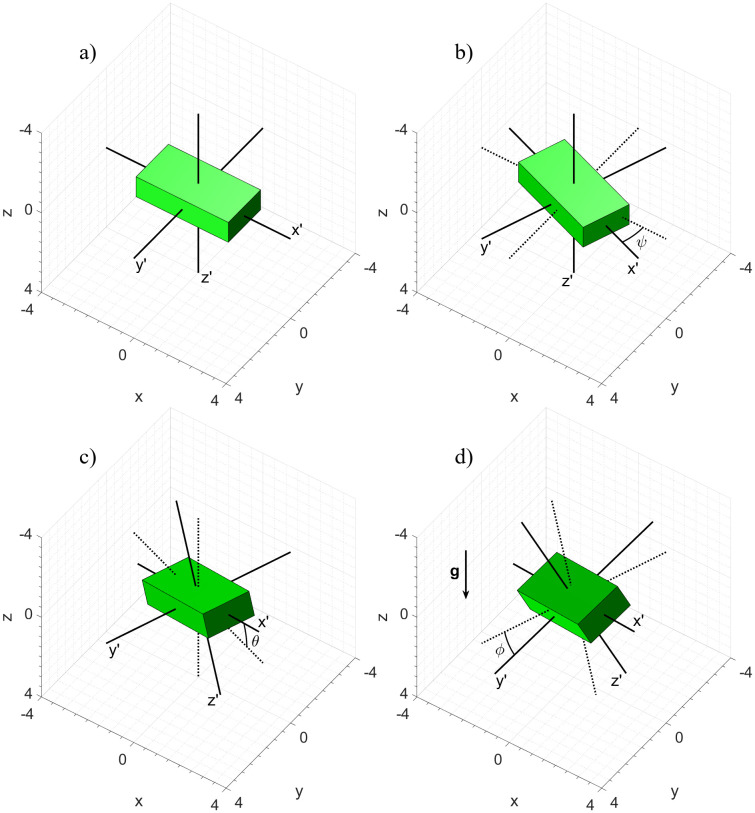
The sequence of Euler angles illustrated using a cuboid. The coordinates in the laboratory frame are denoted by (*x*,*y*,*z*) and the coordinates in the body frame are denoted by $\left(x^{\prime },y^{\prime },z^{\prime }\right)$. The dotted lines correspond to the axes from the previous figure. **a)** Starting orientation for which the body frame coincides with the laboratory frame. **b)** Rotation about the $z^{\prime }$ axis by the angle ψ. **c)** Rotation about the new $y^{\prime }$ axis by the angle θ. **d)** Rotation about the new $x^{\prime }$ axis by the angle ϕ.

To specify the orientation of the object, and with complete generality, we can use the familiar Euler angles [28,29], which we find particularly effective in our studies. The Euler convention we employ is that used in aerospace engineering, where the angles are referred to as yaw, pitch, and roll [28,29]. The sequence of Euler angles is illustrated with a cuboid in Fig 1. The utilization of a cuboid is for illustrative purposes only. Any object with three mutually perpendicular planes of symmetry can be considered. The coordinates in the laboratory frame are denoted by (*x*,*y*,*z*) and the coordinates in the body frame are denoted by $\left(x^{\prime },y^{\prime },z^{\prime }\right)$, along which the principal axes lie. Starting with the orientation shown in Fig 1a, there is first a rotation about the $z^{\prime }$ axis by the angle ψ shown in Fig 1b, then a rotation about the new $y^{\prime }$ axis by the angle θ shown in Fig 1c, and finally a rotation about the new $x^{\prime }$ axis by the angle ϕ shown in Fig 1d. The transformation from the laboratory frame to the body frame is provided by application of rotation matrices as follows [28,29]:


$$\begin{array}{c} \left(\begin{array}{c} x^{\prime }\\ y^{\prime }\\ z^{\prime } \end{array}\right)={𝐑}_{x^{\prime }}(\phi )\cdot {𝐑}_{y^{\prime }}(\theta )\cdot {𝐑}_{z^{\prime }}(\psi )\left(\begin{array}{c} x\\ y\\ z \end{array}\right), \end{array}$$ (3)

where


$$\begin{array}{c} {𝐑}_{x^{\prime }}(\phi )=\left(\begin{array}{ccc} 1 & 0 & 0\\ 0 & \cos\phi & \sin\phi \\ 0 & -\sin\phi & \cos\phi \end{array}\right), \end{array}$$ (4)

$$\begin{array}{c} {𝐑}_{y^{\prime }}(\theta )=\left(\begin{array}{ccc} \cos\theta & 0 & -\sin\theta \\ 0 & 1 & 0\\ \sin\theta & 0 & \cos\theta \end{array}\right), \end{array}$$ (5)

$$\begin{array}{c} {𝐑}_{z^{\prime }}(\psi )=\left(\begin{array}{ccc} \cos\psi & \sin\psi & 0\\ -\sin\psi & \cos\psi & 0\\ 0 & 0 & 1 \end{array}\right), \end{array}$$ (6)

and the subscripts refer to the axes around which the rotation occurs by the angles indicated in parentheses. Conversely, the transformation from the body frame to the laboratory frame is provided by application of the inverse rotation matrices as follows:


$$\begin{array}{c} \left(\begin{array}{c} x\\ y\\ z \end{array}\right)={𝐑}_{z^{\prime }}^{𝐓}(\psi )\cdot {𝐑}_{y^{\prime }}^{𝐓}(\theta )\cdot {𝐑}_{x^{\prime }}^{𝐓}(\phi )\left(\begin{array}{c} x^{\prime }\\ y^{\prime }\\ z^{\prime } \end{array}\right), \end{array}$$ (7)

where the superscript refers to the transpose (and inverse) of the matrices given in Eqs (4), (5), and (6). The gravitational field – and the positive *z* axis – are directed downward as illustrated in Fig 1d. The downward force is the aforementioned effective weight $W_{eff}$, which is assumed to be positive.

To obtain an expression for the velocity of the cuboid shown in Fig 1d, we note that the mobility matrix is diagonal in a coordinate frame in which the principal axes of the cuboid coincide with the axes of that frame as shown in Fig 1a. Therefore, we first transform the external force $W_{eff}$ acting on the cuboid of Fig 1d from the laboratory frame to the body frame by an application of the rotation matrices of Eq (4), (5), and (6) giving


$$\begin{array}{c} \left(\begin{array}{c} F_{x^{\prime }}\\ F_{y^{\prime }}\\ F_{z^{\prime }} \end{array}\right)={𝐑}_{x^{\prime }}(\phi )\cdot {𝐑}_{y^{\prime }}(\theta )\cdot {𝐑}_{z^{\prime }}(\psi )\left(\begin{array}{c} 0\\ 0\\ W_{eff} \end{array}\right)=\left(\begin{array}{c} -W_{eff}\sin\theta \\ W_{eff}\cos\theta \sin\phi \\ W_{eff}\cos\theta \cos\phi \end{array}\right). \end{array}$$ (8)

Note that the external force in the body frame is independent of ψ. This is to be expected, since the external force in the laboratory frame is along the *z* axis and rotation about that axis does not affect the behavior of the falling object as the angle ψ is changed. Therefore, without loss of generality we take ψ=0 (unless otherwise noted), since a laboratory frame for which ψ=0 can always be chosen by a suitable rotation about the *z* axis.

Noting again that the mobility matrix is diagonal in the body frame, since the body-frame axes are parallel to the principal axes of the cuboid, we have from Eqs (1) and (8) for the velocity of the object in the body frame:


$$\begin{array}{c} \left(\begin{array}{c} U_{x^{\prime }}\\ U_{y^{\prime }}\\ U_{z^{\prime }} \end{array}\right)=\left(\begin{array}{ccc} \mu_{x^{\prime }x^{\prime }} & 0 & 0\\ 0 & \mu_{y^{\prime }y^{\prime }} & 0\\ 0 & 0 & \mu_{z^{\prime }z^{\prime }} \end{array}\right)\left(\begin{array}{c} -W_{eff}\sin\theta \\ W_{eff}\cos\theta \sin\phi \\ W_{eff}\cos\theta \cos\phi \end{array}\right)=\left(\begin{array}{c} -\mu_{x^{\prime }x^{\prime }}W_{eff}\sin\theta \\ \mu_{y^{\prime }y^{\prime }}W_{eff}\cos\theta \sin\phi \\ \mu_{z^{\prime }z^{\prime }}W_{eff}\cos\theta \cos\phi \end{array}\right). \end{array}$$ (9)

Applying the rotation matrices ${𝐑}_{z^{\prime }}^{𝐓}(\psi )\cdot {𝐑}_{y^{\prime }}^{𝐓}(\theta )\cdot {𝐑}_{x^{\prime }}^{𝐓}(\phi )$ from Eqs (7) to (9) to transform from the body frame back to the laboratory frame, we find for the velocity components of the object in the laboratory frame:


$$\begin{array}{c} \left(\begin{array}{c} U_{x}(\phi ,\theta ,0)\\ U_{y}(\phi ,\theta ,0)\\ U_{z}(\phi ,\theta ,0) \end{array}\right)=\left(\begin{array}{c} W_{eff}(\mu_{z^{\prime }z^{\prime }}\cos^{2}\phi +\mu_{y^{\prime }y^{\prime }}\sin^{2}\phi -\mu_{x^{\prime }x^{\prime }})\cos\theta \sin\theta \\ W_{eff}(\mu_{y^{\prime }y^{\prime }}-\mu_{z^{\prime }z^{\prime }})\cos\theta \cos\phi \sin\phi \\ W_{eff}(\mu_{z^{\prime }z^{\prime }}\cos^{2}\phi +\mu_{y^{\prime }y^{\prime }}\sin^{2}\phi )\cos^{2}\theta +W_{eff}\mu_{x^{\prime }x^{\prime }}\sin^{2}\theta \end{array}\right). \end{array}$$ (10)

Now define the speed of the object oriented so that it is falling along the $x^{\prime }$ direction as *U*_1_, along the $y^{\prime }$ direction as *U*_2_, and along the $z^{\prime }$ direction as *U*_3_. Then we have for (ϕ,θ,ψ)=(0,π/2,0)


$$\begin{array}{c} \left(\begin{array}{c} U_{x}(0,\pi /2,0)\\ U_{y}(0,\pi /2,0)\\ U_{z}(0,\pi /2,0) \end{array}\right)=\left(\begin{array}{c} 0\\ 0\\ W_{eff}\mu_{x^{\prime }x^{\prime }} \end{array}\right)=\left(\begin{array}{c} 0\\ 0\\ U_{1} \end{array}\right), \end{array}$$ (11)

for (ϕ,θ,ψ)=(π/2,0,0)


$$\begin{array}{c} \left(\begin{array}{c} U_{x}(\pi /2,0,0)\\ U_{y}(\pi /2,0,0)\\ U_{z}(\pi /2,0,0) \end{array}\right)=\left(\begin{array}{c} 0\\ 0\\ W_{eff}\mu_{y^{\prime }y^{\prime }} \end{array}\right)=\left(\begin{array}{c} 0\\ 0\\ U_{2} \end{array}\right), \end{array}$$ (12)

and for (ϕ,θ,ψ)=(0,0,0)


$$\begin{array}{c} \left(\begin{array}{c} U_{x}(0,0,0)\\ U_{y}(0,0,0)\\ U_{z}(0,0,0) \end{array}\right)=\left(\begin{array}{c} 0\\ 0\\ W_{eff}\mu_{z^{\prime }z^{\prime }} \end{array}\right)=\left(\begin{array}{c} 0\\ 0\\ U_{3} \end{array}\right). \end{array}$$ (13)

Therefore, we may replace $W_{eff}\mu_{x^{\prime }x^{\prime }}$, $W_{eff}\mu_{y^{\prime }y^{\prime }}$, and $W_{eff}\mu_{x^{\prime }x^{\prime }}$, with *U*_1_, *U*_2_, and *U*_3_, respectively, in Eq (10) giving


$$\begin{array}{c} \left(\begin{array}{c} U_{x}(\phi ,\theta ,0)\\ U_{y}(\phi ,\theta ,0)\\ U_{z}(\phi ,\theta ,0) \end{array}\right)=\left(\begin{array}{c} (U_{3}\cos^{2}\phi +U_{2}\sin^{2}\phi -U_{1})\cos\theta \sin\theta \\ (U_{2}-U_{3})\cos\theta \cos\phi \sin\phi \\ (U_{3}\cos^{2}\phi +U_{2}\sin^{2}\phi )\cos^{2}\theta +U_{1}\sin^{2}\theta \end{array}\right). \end{array}$$ (14)

Eq (14) is a *general formula*, and a main result of the present paper, giving the components of velocity for any object of uniform density and arbitrary orientation far from any boundaries and with three mutually perpendicular planes of symmetry falling through a viscous fluid in the Stokes limit in terms of the speeds of the object falling through the fluid in a direction along each of its three mutually perpendicular principal axes.

Noting that the drag force of the fluid on the object is equal and opposite to the effective weight of the object we have


$$\begin{array}{c} W_{eff}=F_{drag}=\zeta_{1}U_{1}=\zeta_{2}U_{2}=\zeta_{3}U_{3}, \end{array}$$ (15)

where $W_{eff}$ and $F_{drag}$ are magnitudes of those forces, and $\zeta_{1}$, $\zeta_{2}$, and $\zeta_{3}$ are the drag coefficients along the $x^{\prime }$, $y^{\prime }$, and $z^{\prime }$ directions, respectively. Therefore, Eq (13), as well as subsequent equations, may be written in terms of the drag coefficients $\zeta_{1}$, $\zeta_{2}$, and $\zeta_{3}$ by replacing *U*_1_, *U*_2_, and *U*_3_ with $W_{eff}/\zeta_{1}$, $W_{eff}/\zeta_{2}$, and $W_{eff}/\zeta_{3}$, respectively. For future reference we also define the body lengths along the principal axes $x^{\prime }$, $y^{\prime }$, and $z^{\prime }$ as *L*_1_, *L*_2_, and *L*_3_, respectively. To summarize, given the speeds of the falling object along each of its three principal axes, or the effective weight and drag coefficients along each of its principal axes, the three components of velocity, including the sideways components, have been determined for an arbitrary orientation of the object.

## 3. Results

The general formula Eq (14) applies to all the shapes we consider. So, in the following we can describe them in an integrated approach as seen in the set of figures shown below.

### 3.1. Examples for the general case

**Fig 2:**
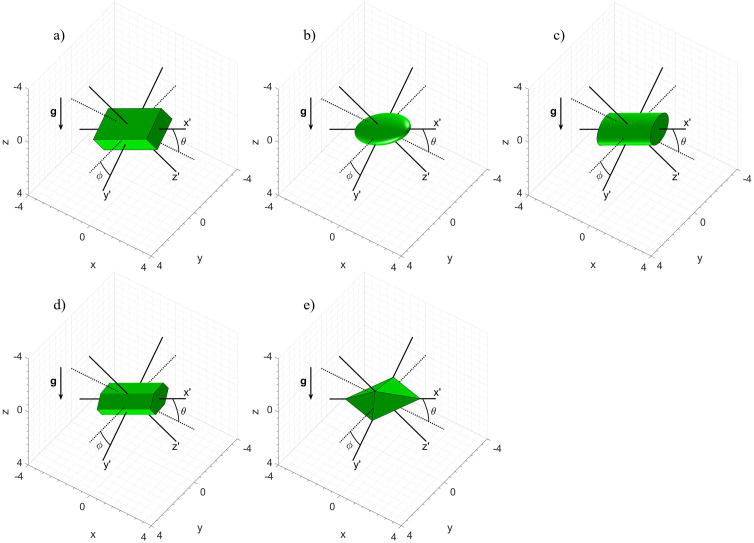
Illustrations for the general case of a falling object with three mutually perpendicular planes of symmetry. Here, the drag coefficients along the three principal axes are all different from one another. The objects are a) a cuboid (or rectangular prism), b) an ellipsoid, c) an elliptic cylinder, d) a hexagonal prism, and e) an octahedron. We take the lengths *L*_1_, *L*_2_, and *L*_3_ along the principal axes $x^{\prime }$, $y^{\prime }$, and $z^{\prime }$, respectively, such that $L_{1}=2L_{2}=4L_{3}$. The sequence of Euler angles is given in section 3.1 in the text. The velocity of the objects is described by Eqs (14) and (15).

Here, we provide examples for the general case, that is, objects with three mutually perpendicular planes of symmetry for which the drag coefficients along each of its three principal axes are not equal. Some examples are a cuboid (or rectangular prism), an ellipsoid, an elliptic cylinder, a hexagonal prism, and an octahedron. These objects are illustrated in Fig 2. The motion of all these objects when falling through a fluid in the Stokes limit are described by Eqs (14) and (15). Given the speeds *U*_1_, *U*_2_, and *U*_3_ of the object falling along each of its principal axes, or the effective weight and drag coefficients along each of its principal axes, the three components of velocity in the laboratory frame, including the sideways components, are determined for an arbitrary orientation of the object, where the orientation is given in terms of the Euler angles θ and ϕ. Note that we need only consider the angles θ and ϕ, since we take ψ=0 without loss of generality as discussed after Eq (8). The sequence of Euler angles is rotation about the original $y^{\prime }$ axis (indicated by the dotted line coinciding with the *y* axis) by angle θ, and then rotation about the new $x^{\prime }$ axis by angle ϕ.

#### 3.1.1. An application to experiment.

We now apply Eq (14) to an experimental system [3]. Aluminum blocks machined to a high tolerance (±0.0003cm) in the shape of cuboids were released in a viscous oil along each of their three principal axes, and their settling velocities measured. The dimensions of the cuboids were on the order of centimeters, but because of the high viscosity of the oil, the Stokes flow approximation was valid. We chose one of these cuboids for which the velocities along each principal axis were significantly different from one another. The dimensions of this cuboid were $L_{1}=2.032\;cm$, $L_{2}=0.511\;cm$, and $L_{3}=0.130\;cm$.

The viscous drag force on a sphere is given by the well-known Stokes Law


$$\begin{array}{c} F_{drag}=3\pi \eta dU, \end{array}$$ (16)

where *d* is the diameter of the sphere and *U* is the speed of the sphere relative to the fluid.

**Fig 3:**
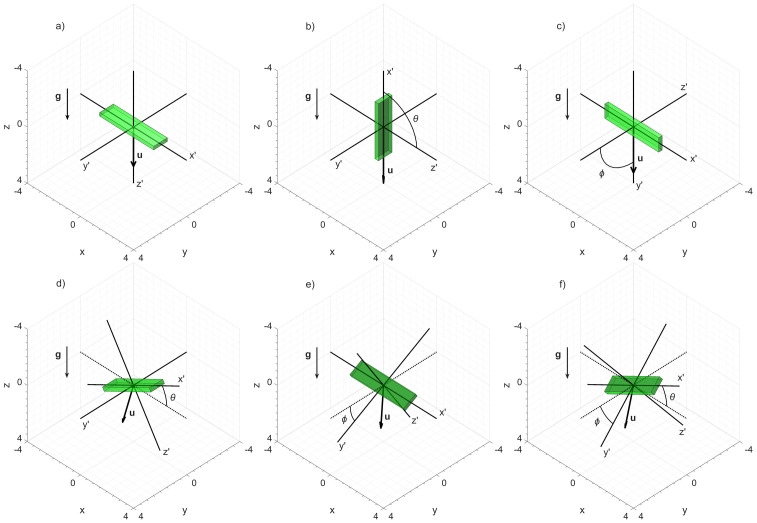
Illustrations showing an object from experiment and the velocity vector for various orientations. The object is a cuboid from an experiment described in [3]. The dimensions of the object are $L_{1}=2.032\;cm$, $L_{2}=0.511\;cm$, and $L_{3}=0.130\;cm$. The settling speed measured from experiment along each of its principal axes are *U*_1_ = 1, *U*_2_ = 0.818, and *U*_3_ = 0.704, where the speed is scaled to that parallel to *L*_1_. The velocity of the object for arbitrary orientation is predicted by Eq (14). The respective triplets (ϕ,θ,ψ), $\left(U_{x},U_{y},U_{z}\right)$ are for **a)** (0,0,0), (0,0,0.704); **b)** (0,π/2,0),(0,0,1); **c)** (π/2,0,0),(0,0,0.818); **d)** (0,π/6,0),(−0.128,0,0.778); **e)** (π/6,0,0),(0,0.050,0.733); **f)** (π/6,π/6,0),(−0.116,0.043,0.799).

For a nonspherical object the viscous drag force may be written as


$$\begin{array}{c} F_{drag}=\frac{3\pi \eta d_{s}U}{K}, \end{array}$$ (17)

where $d_{s}$ is the diameter of a sphere with the same volume as that of the object and *K* is the shape resistance factor. From this equation it is seen that the speed of the object is proportional to *K*. We define *K*_1_, *K*_2_, and *K*_3_ as the shape resistance factors when the object is falling parallel to *L*_1_, *L*_2_, and *L*_3_, respectively. From [3] we find *K*_1_ = 0.804, *K*_2_ = 0.658, and *K*_3_ = 0.566. Scaling the speed of the object to the speed when falling parallel to *L*_1_, we then find *U*_1_ = 1, $U_{2}=K_{2}/K_{1}=0.818$, and $U_{3}=K_{3}/K_{1}=0.704$. Since we now have the speed of this object falling along each of its principal axes, we may apply Eq (14) to give the velocity of the object, including sideways components, for arbitrary orientation. Fig 3 shows the velocity vector for this object for various orientations.

Fig 3a–3c show the experimentally measured velocities when the cuboid is falling parallel to each of its principal axes, the speed parallel to the long axis being the largest (presenting the least cross-sectional area to the direction of motion, Fig 3b), and the speed parallel to the short axis being the smallest (presenting the maximum cross-sectional area to the direction of motion, Fig 3a). When the object makes an angle θ=π/6 about the $y^{\prime }$ axis it has a sideways drift in the −x direction (Fig 3d). When the object makes an angle ϕ=π/6 about the $x^{\prime }$ axis it has a sideways drift in the + *y* direction (Fig 3e). When it makes an angle θ=π/6 about the $y^{\prime }$ axis and an angle ϕ=π/6 about the $x^{\prime }$ axis it will have a sideways drift in both the −x direction and the + *y* direction (Fig 3f). The values for the velocity components determined by Eq (14) are given in the Fig 3 caption.

Although Eq (14) will be accurate for macroscopic objects with three mutually perpendicular planes of symmetry in a highly viscous fluid, such as the cuboids of [3], smaller objects on the order of a micron or smaller in size will exhibit significant rotational Brownian motion, Therefore, such an object will not maintain its orientation as it settles in the fluid but it will rather rotate randomly as it settles. In this case the settling velocity of the object will simply be the average of the settling velocities along each principal axis, that is, $U_{z}=(U_{1}+U_{2}+U_{3})/3$ and $U_{x}=U_{y}=0$ [3].

We may rigorously verify this formula by averaging the velocity over all Euler angles. Since we are averaging over all Euler angles, we need to include a rotation of ψ about the *z*-axis and thus apply all three rotation matrices to Eq (9) as


$$\begin{array}{c} U(\phi ,\theta ,\psi )={𝐑}_{z^{\prime }}^{𝐓}(\psi )\cdot {𝐑}_{y^{\prime }}^{𝐓}(\theta )\cdot {𝐑}_{x^{\prime }}^{𝐓}(\phi )\left(\begin{array}{c} -\mu_{1}W_{eff}\sin\theta \\ \mu_{2}W_{eff}\cos\theta \sin\phi \\ \mu_{3}W_{eff}\cos\theta \cos\phi \end{array}\right), \end{array}$$ (18)

where we define $\mu_{1}=\mu_{x^{\prime }x^{\prime }}$, $\mu_{2}=\mu_{y^{\prime }y^{\prime }}$, and $\mu_{3}=\mu_{z^{\prime }z^{\prime }}$. To integrate over all the Euler angles we use the Haar measure (the volume element for the rotation group SO(3)) dΩ=cosθdθdϕdψ where θ∈[−π/2,π/2], ϕ∈[0,2π], and ψ∈[0,2π] [30]. The cosθ term results from the probability of being near the equator (θ=0) being larger than the probability of being near the poles (θ=±π/2). The average of Eq (18) over all Euler angles is given by


$$\begin{array}{c} U_{avg}=\frac{\int d\Omega \;U(\phi ,\theta ,\psi )}{\int d\Omega }=\frac{1}{8\pi^{2}}\int d\Omega \;U(\phi ,\theta ,\psi ). \end{array}$$ (19)

Performing the integral then gives


$$\begin{array}{c} U_{\mathrm{avg}}=\left(\begin{array}{c} 0\\ 0\\ \frac{1}{3}W_{eff}(\mu_{1}+\mu_{2}+\mu_{3}) \end{array}\right)=\left(\begin{array}{c} 0\\ 0\\ \frac{1}{3}(U_{1}+U_{2}+U_{3}) \end{array}\right), \end{array}$$ (20)

which is the expected result. Matlab was used to calculate the integrals. Details may be found in the PDF S1 File Euler_average.pdf of the Matlab live script in the supporting information.

### 3.2. Special cases

We consider two special cases. For ϕ=0 we have


$$\begin{array}{c} \left(\begin{array}{c} U_{x}(0,\theta ,0)\\ U_{y}(0,\theta ,0)\\ U_{z}(0,\theta ,0) \end{array}\right)=\left(\begin{array}{c} (U_{3}-U_{1})\cos\theta \sin\theta \\ 0\\ U_{3}\cos^{2}\theta +U_{1}\sin^{2}\theta \end{array}\right), \end{array}$$ (21)

and for θ=0 we have


$$\begin{array}{c} \left(\begin{array}{c} U_{x}(\phi ,0,0)\\ U_{y}(\phi ,0,0)\\ U_{z}(\phi ,0,0) \end{array}\right)=\left(\begin{array}{c} 0\\ (U_{2}-U_{3})\cos\phi \sin\phi \\ U_{3}\cos^{2}\phi +U_{2}\sin^{2}\phi \end{array}\right). \end{array}$$ (22)

Note that these two equations have the same form as functions of the angles. In Eq (21) there is only a rotation about the $y^{\prime }$ axis by an angle θ and in Eq (22) there is only a rotation about the $x^{\prime }$ axis by an angle ϕ. Therefore, we would expect that the angular functions are the same. It should be noted that in the case of the rotation about the $y^{\prime }$ axis for 0<θ<π/2, the sideways motion is in the −x direction, assuming $U_{1}>U_{3}$, and for the rotation about the $x^{\prime }$ axis for 0<ϕ<π/2, the sideways motion is in the + *y* direction, assuming $U_{2}>U_{3}$, as would be the case for the cuboid illustrated in Fig 1. It is important to note that when discussing rotations about the primed axes here and in the remainder of the paper, we mean *rotations that prepare the orientation of the object for its release*. Once released, *the object maintains its orientation as it falls through the fluid with constant velocity*.

#### 3.2.1. The drag coefficients along two axes are equal.

**Fig 4:**
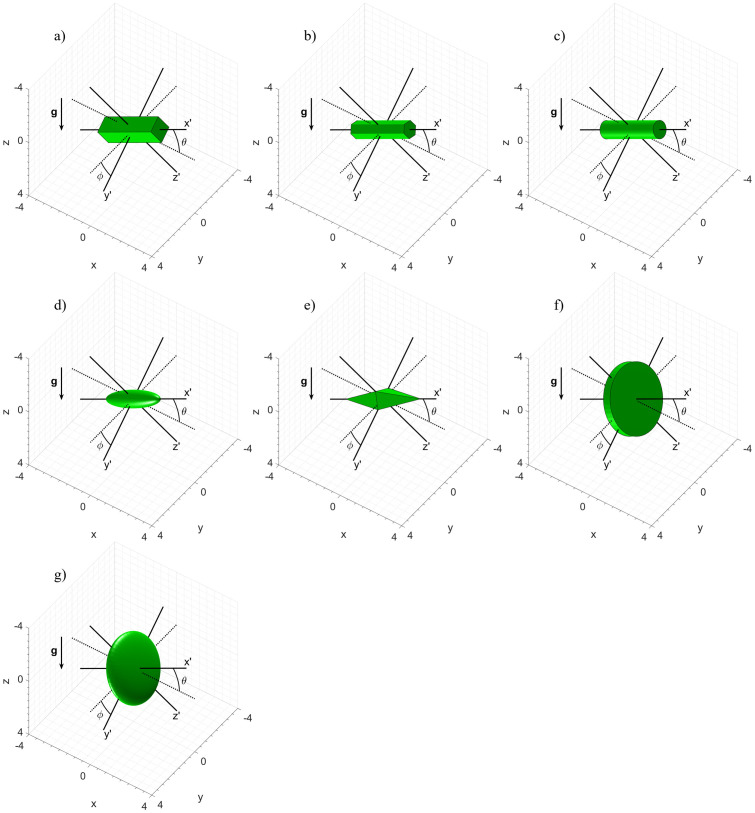
Illustration of a falling object for the special case where the drag coefficients along two axes are equal. Here the drag coefficients along the $y^{\prime }$ and $z^{\prime }$ directions are equal. The objects are a) a rod with square cross section, b) a rod with a regular hexagonal cross section, c) a rod with a circular cross section, d) a prolate spheroid, e) a prolate octahedron, f) a circular disk, and g) an oblate spheroid. Prolate refers to $L_{1}>L_{2}=L_{3}$ and oblate refers to $L_{1}<L_{2}=L_{3}$. The sequence of Euler angles is given in section 3.1 in the text. The velocity of the falling object is given by Eq (17) and is independent of the angle ϕ about the $x^{\prime }$ axis. Therefore, as the object falls its velocity is independent of its orientation about the $x^{\prime }$ axis.

Now consider the case where $U_{3}=U_{2}$, which would correspond to the drag coefficient along the $y^{\prime }$ and $z^{\prime }$ directions being equal. In this case Eq (14) reduces to


$$\begin{array}{c} \left(\begin{array}{c} U_{x}(\phi ,\theta ,0)\\ U_{y}(\phi ,\theta ,0)\\ U_{z}(\phi ,\theta ,0) \end{array}\right)=\left(\begin{array}{c} (U_{3}-U_{1})\cos\theta \sin\theta \\ 0\\ U_{3}\cos^{2}\theta +U_{1}\sin^{2}\theta \end{array}\right), \end{array}$$ (23)

which is identical to Eq (21), except that here ϕ is arbitrary. Note that this equation can also be written in terms of the drag coefficients by using Eq (15). These equations can be applied to a rod with square cross section pointing along the $x^{\prime }$ direction as illustrated in Fig 4a. Since Eq (23) is independent of ϕ, the velocity of the falling rod is independent of its orientation about the $x^{\prime }$ axis. Eq (23) may also be applied to a rod with other cross sections such as a regular hexagonal cross section and a circular cross section as illustrated in Fig 4b and 4c, respectively. In addition, this equation can be applied to a prolate spheroid and an octahedron for which two of the principal axes are the same as illustrated in Fig 4d and 4e, respectively, as well as a circular disk and an oblate spheroid as illustrated in Fig 4f and 4g, respectively. Eq (23) can be shown to be equivalent to Eqs (34) and (35) derived in [2] for the case of an obliquely falling cylindrical rod with circular cross section, for which the angle θ is measured from the vertical, after performing the transformation θ→θ+π/2 and using the double angle formulas for the sine and cosine functions. Note also that the equations for a cylindrical rod with circular cross section derived in [2] may be applied to any of the objects illustrated in Fig 4.

We consider the prolate and oblate spheroids illustrated in Fig 4d and 4g, for which there are exact analytic solutions. A spheroid is created by rotating an ellipse about one of its axes of symmetry – about the major axis for a prolate spheroid and about the minor axis for an oblate spheroid. Analytic equations for the drag coefficients for both axial and transverse flow for a prolate and oblate spheroid may be found in [31]. They may also be used to find the drag coefficients for a long thin rod and for a thin disk [32].

A **prolate spheroid** is now considered. Here we take the symmetry axis to be in the $x^{\prime }$ direction, as shown in Fig 4d for a prolate spheroid ($L_{1}>L_{2}=L_{3}$). Recall that *L*_1_, *L*_2_, and *L*_3_ are the body lengths along the $x^{\prime }$, $y^{\prime }$, and $z^{\prime }$ directions, respectively. For θ=π/2, the spheroid is oriented such that it falls in a direction parallel to its symmetry axis, and the magnitudes of the drag force and effective weight are given by [31]


$$\begin{array}{c} F_{drag}=W_{eff}=8\pi \eta U_{1}L_{1}e_{p}^{3}{\left[(1+e_{p}^{2})\ln\left(\frac{1+e_{p}}{1-e_{p}}\right)-2e_{p}\right]}^{-1}, \end{array}$$ (24)

where the eccentricity of the prolate spheroid is given by $e_{p}=\sqrt{1-{L_{3}}^{2}/{L_{1}}^{2}}$, η is the dynamic viscosity of the fluid, *U*_1_ is the speed of the spheroid falling along the $x^{\prime }$ direction, and, since $L_{1}>L_{3}$, *L*_1_ and *L*_3_ are the lengths of the major and minor axes, respectively. Solving for *U*_1_ gives for the *speed of a prolate spheroid falling parallel to its long axis*


$$\begin{array}{c} U_{1}=\frac{W_{eff}}{8\pi \eta L_{1}e_{p}^{3}}\left[(1+e_{p}^{2})\ln\left(\frac{1+e_{p}}{1-e_{p}}\right)-2e_{p}\right]. \end{array}$$ (25)

For the limit $L_{3}/L_{1}\to 0$ we have for the *speed of a long thin prolate spheroid falling parallel to its long axis* ($L_{3}\ll L_{1}$) the result


$$\begin{array}{c} U_{1}=\frac{W_{eff}}{2\pi \eta L_{1}}\ln\frac{L_{1}}{L_{3}}. \end{array}$$ (26)

For θ=0, the spheroid is oriented such that it falls in a direction perpendicular to its symmetry axis, and the magnitudes of the drag force and effective weight are given by [31]


$$\begin{array}{c} F_{drag}=W_{eff}=16\pi \eta U_{3}L_{1}e_{p}^{3}{\left[(3e_{p}^{2}-1)\ln\left(\frac{1+e_{p}}{1-e_{p}}\right)+2e_{p}\right]}^{-1}, \end{array}$$ (27)

where *U*_3_ is the speed of the spheroid falling along the $z^{\prime }$ direction. Solving for *U*_3_ gives for *the speed of a prolate spheroid falling perpendicular to its long axis*


$$\begin{array}{c} U_{3}=\frac{W_{eff}}{16\pi \eta L_{1}e_{p}^{3}}\left[(3e_{p}^{2}-1)\ln\left(\frac{1+e_{p}}{1-e_{p}}\right)+2e_{p}\right]. \end{array}$$ (28)

Again, we consider the limit $L_{3}/L_{1}\to 0$ for which the *speed of a long thin prolate spheroid falling perpendicular to its long axis* ($L_{3}\ll L_{1}$) is given by


$$\begin{array}{c} U_{3}=\frac{W_{eff}}{4\pi \eta L_{1}}\ln\frac{L_{1}}{L_{3}}. \end{array}$$ (29)

The speeds *U*_1_ and *U*_3_ from Eqs (25) and (28), respectively, may be substituted into Eq (23) to give the horizontal $U_{x}$ and vertical $U_{z}$ components of velocity of a falling prolate spheroid for an arbitrary angle θ measured from the horizontal.

Also, the speeds *U*_1_ and *U*_3_ from Eqs (26) and (29), respectively, may be substituted into Eq (23) to give the horizontal $U_{x}$ and vertical $U_{z}$ components of velocity of a falling long thin prolate spheroid for an arbitrary angle θ measured from the horizontal. Since the form of Eqs (26) and (29) are particularly simple, we do the substitution and obtain


$$\begin{array}{c} \left(\begin{array}{c} U_{x}(\phi ,\theta ,0)\\ U_{y}(\phi ,\theta ,0)\\ U_{z}(\phi ,\theta ,0) \end{array}\right)=\frac{W_{eff}}{4\pi \eta L_{1}}\ln\frac{L_{1}}{L_{3}}\left(\begin{array}{c} -\cos\theta \sin\theta \\ 0\\ \cos^{2}\theta +2\sin^{2}\theta \end{array}\right). \end{array}$$ (30)

As θ is changed from 0 to π/2, the orientation of the spheroid changes from horizontal to vertical, and the vertical component of velocity changes from 1 to 2, scaled to the speed of the spheroid with a horizontal orientation. This is consistent with a long thin rod falling twice as fast in a direction parallel to its length as compared to the rod falling in a direction perpendicular to its length [2,23–25].

An **oblate spheroid** is now considered. For an oblate spheroid ($L_{1}<L_{2}=L_{3}$) the symmetry axis is again taken to be in the $x^{\prime }$ direction as shown in Fig 4g. For θ=π/2, the spheroid is oriented such that it falls in a direction parallel to its symmetry axis, and the magnitudes of the drag force and effective weight are given by [31]


$$\begin{array}{c} F_{drag}=W_{eff}=\frac{4\pi \eta U_{1}L_{3}e_{o}^{3}}{e_{o}{\left(1-e_{0}^{2}\right)}^{1/2}-(1-2e_{o}^{2})\sin^{-1}e_{o}}, \end{array}$$ (31)

where the eccentricity of the oblate spheroid is given by $e_{o}=\sqrt{1-{L_{1}}^{2}/{L_{3}}^{2}}$, η is the dynamic viscosity of the fluid, *U*_1_ is the speed of the spheroid falling along the $x^{\prime }$ direction, and, since $L_{1}<L_{3}$, *L*_1_ and *L*_3_ are now the lengths of the minor and major axes, respectively. Solving for *U*_1_ gives for the *speed of an oblate spheroid falling parallel to its short axis*


$$\begin{array}{c} U_{1}=\frac{W_{eff}}{4\pi \eta L_{3}e_{o}^{3}}\left[e_{o}{\left(1-e_{0}^{2}\right)}^{1/2}-(1-2e_{o}^{2})\sin^{-1}e_{o}\right]. \end{array}$$ (32)

Consider the limit $L_{1}/L_{3}\to 0$. We then have for the *speed of a thin oblate spheroid falling parallel to its short axis* ($L_{1}\ll L_{3}$)


$$\begin{array}{c} U_{1}=\frac{W_{eff}}{8\eta L_{3}}. \end{array}$$ (33)

For θ=0, the spheroid is oriented such that it falls in a direction perpendicular to its symmetry axis, and the magnitudes of the drag force and effective weight are given by [31]


$$\begin{array}{c} F_{drag}=W_{eff}=\frac{8\pi \eta U_{3}L_{3}e_{o}^{3}}{-e_{o}{\left(1-e_{0}^{2}\right)}^{1/2}+(1+2e_{o}^{2})\sin^{-1}e_{o}}, \end{array}$$ (34)

where *U*_3_ is the speed of the spheroid falling along the $z^{\prime }$ direction. Solving for *U*_3_ gives for the *speed of an oblate spheroid falling perpendicular to its short axis*


$$\begin{array}{c} U_{3}=\frac{W_{eff}}{8\pi \eta L_{3}e_{o}^{3}}\left[-e_{o}{\left(1-e_{0}^{2}\right)}^{1/2}+(1+2e_{o}^{2})\sin^{-1}e_{o}\right]. \end{array}$$ (35)

Again, consider the limit of $L_{1}/L_{3}\to 0$. We then have for the speed of a thin oblate spheroid falling perpendicular to its short axis ($L_{1}\ll L_{3}$)


$$\begin{array}{c} U_{3}=\frac{3W_{eff}}{16\eta L_{3}}. \end{array}$$ (36)

The speeds *U*_1_ and *U*_3_ from Eqs (32) and (35), respectively, may be substituted into Eq (23) to give the horizontal $U_{x}$ and vertical $U_{z}$ components of velocity of a falling oblate spheroid for an arbitrary angle θ measured from the horizontal.

Also, the speeds *U*_1_ and *U*_3_ from Eqs (33) and (36) respectively, may be substituted into Eq (23) to give the horizontal $U_{x}$ and vertical $U_{z}$ components of velocity of a falling thin oblate spheroid for an arbitrary angle θ measured from the horizontal. Since the form of Eqs (33) and (36) are particularly simple, we do the substitution and obtain


$$\begin{array}{c} \left(\begin{array}{c} U_{x}(\phi ,\theta ,0)\\ U_{y}(\phi ,\theta ,0)\\ U_{z}(\phi ,\theta ,0) \end{array}\right)=\frac{3W_{eff}}{16\eta L_{3}}\left(\begin{array}{c} \cos\theta \sin\theta \\ 0\\ \cos^{2}\theta +(2/3)\sin^{2}\theta \end{array}\right). \end{array}$$ (37)

As θ is changed from 0 to π/2, the orientation of the symmetry axis of the spheroid changes from a horizontal to a vertical position, and the vertical component of velocity changes from 1 to 2/3, scaled to the speed of the spheroid with a horizontal orientation. This is consistent with the speed of a thin circular disk falling parallel to its symmetry axis being 2/3 the speed of the disk falling perpendicular to its symmetry axis [28,29].

#### 3.2.2. The drag coefficients along all three axes are equal.

**Fig 5:**
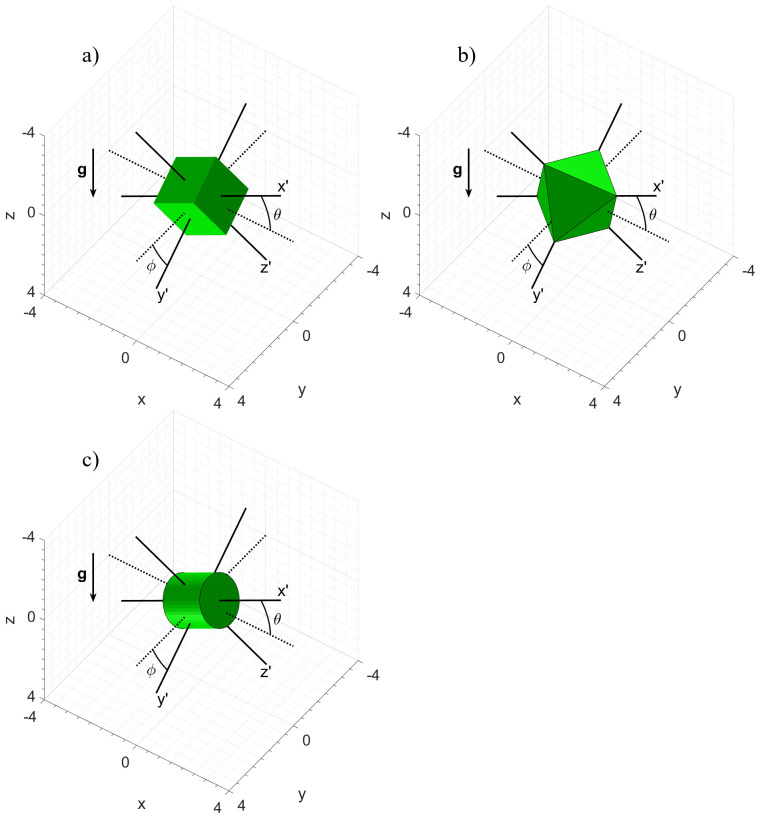
Illustration of a falling object for the special case where the drag coefficients along all three axes are equal. Here, the drag coefficients along the $x^{\prime }$, $y^{\prime }$, and $z^{\prime }$ directions are all equal. The objects are a) a cube, b) a regular octahedron, and c) a cylinder with the appropriate aspect ratio. The sequence of Euler angles is given in section 3.1 in the text. The velocity of the falling object is given by Eq (38) and is independent of the angles ϕ about the $x^{\prime }$ axis and θ about the $y^{\prime }$ axis. Therefore, the object falls straight down with speed *U*_3_ regardless of its orientation.

Now consider the case where the drag coefficient along each of its principal axes are equal. Examples are a cube and a regular octahedron, as illustrated in Fig 5a and 5b, respectively. Therefore, $U_{1}=U_{2}=U_{3}$ in Eq (14) and we find


$$\begin{array}{c} \left(\begin{array}{c} U_{x}(\phi ,\theta ,0)\\ U_{y}(\phi ,\theta ,0)\\ U_{z}(\phi ,\theta ,0) \end{array}\right)=\left(\begin{array}{c} 0\\ 0\\ U_{3} \end{array}\right), \end{array}$$ (38)

showing that the cube and octahedron indeed fall straight down with speed *U*_3_ regardless of their orientation. This result is consistent with the analysis of [23], where symmetry arguments were used.

**Fig 6:**
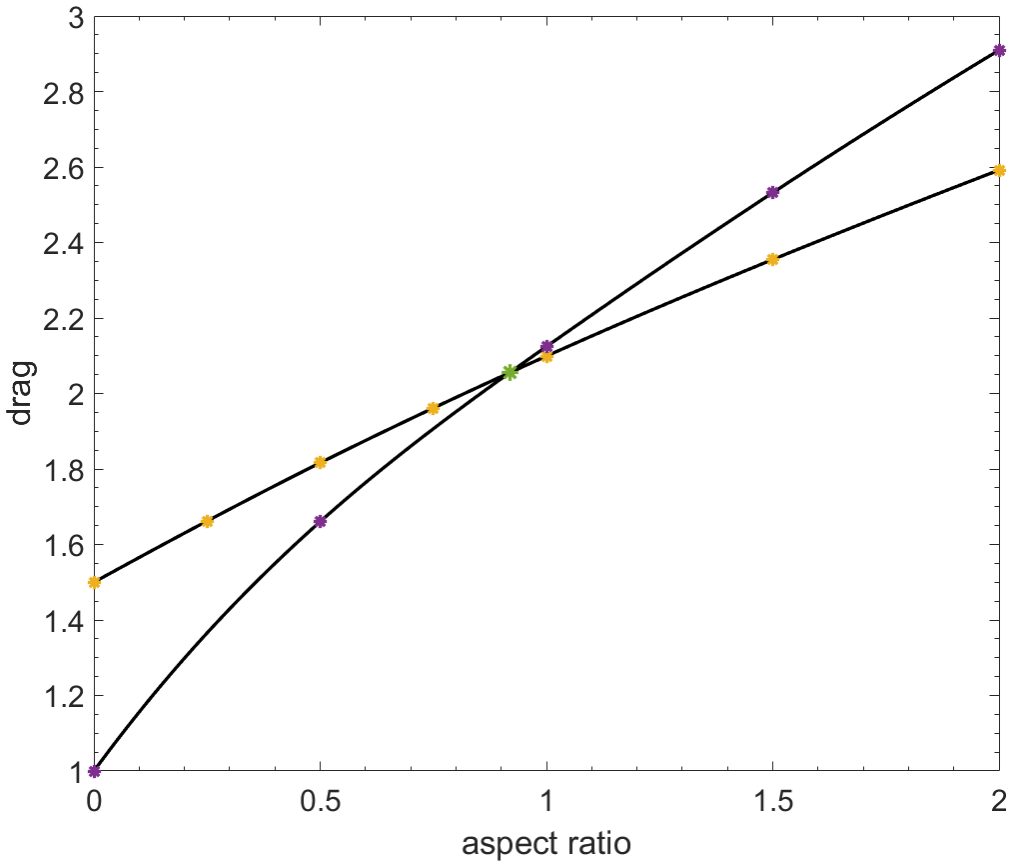
Plot of the drag force on a cylinder for both axial and transverse motion as a function of the aspect ratio (length/diameter). The magenta and red stars correspond to the data from [33]. The curves correspond to cubic spline fits to this data. The green star corresponds to the intersection of these two curves. For this aspect ratio of 0.92, the speed of the falling cylinder is the same for both axial and transverse motion. The drag force is normalized to that for transverse motion of a cylinder with zero aspect ratio.

Although the cases illustrated in Fig 5a and 5b correspond to the lengths of the principal axes being equal, this is generally not the case. To see this consider the rods illustrated in Fig 4b and 4c. As the length of a rod is gradually decreased, at some point the drag coefficients parallel and perpendicular to the rod will be equal. When this is the case, $U_{1}=U_{2}=U_{3}$, and the rod will fall straight down with speed *U*_3_, regardless of orientation. Because of the lack of symmetry, even though $U_{1}=U_{2}=U_{3}$, the lengths along the principal axes are unlikely to be the same. This is illustrated in Fig 5c, which shows a circular cylinder with an aspect ratio length/diameter of 0.92. The value of 0.92 was obtained from [33] by performing cubic spline fits on their data obtained from a bead-on-shell method for both axial and transverse drag for various aspect ratios and determining the intersection of the two curves, as shown in Fig 6. Fig 5c demonstrates that shapes other than the shape of a regular polyhedron can fall straight down regardless of orientation, the only criterion being $U_{1}=U_{2}=U_{3}$.

Throughout this work, we have primarily considered the motion of an object with three mutually perpendicular planes of symmetry. The motivation stems from the next relevant geometrical shapes beyond the simple cylindrical objects first considered in the literature. For such generalizations, the translational motion and rotational motion are completely decoupled from one another, and the object maintains its orientation as it falls through the fluid as noted in section 2. This then is a pivotal factor in the derivation of the general formula Eq (14), along with a uniform density and being far from boundaries. A major simplification arises if an object has a high degree of rotational symmetry, such as a regular polyhedron, it will fall straight down regardless of orientation even if the three mutually perpendicular planes of symmetry criterion is not satisfied. For example, a regular tetrahedron does not satisfy this criterion, yet it does fall straight down regardless of orientation [23].

A similar argument can be made for a falling rod with a polygonal cross section considered in section 3.2.1, where it was noted that the velocity of the falling object is independent of its orientation about its long axis. There it was stated that, to satisfy the three mutually perpendicular planes of symmetry criterion, the cross section of the rod would have two orthogonal lines of symmetry. Any regular polygon with an even number of sides would satisfy this criterion. However, because of the high degree of rotational symmetry, we would expect that the velocity of a falling rod with any regular polygonal cross section, including an odd number of sides, would still satisfy Eq (23) and be independent of its orientation about its long axis.

### 3.3. The deflection angles

The expressions for the deflection angles are as follows. The deflection angle $\alpha_{x}$ in the *x* direction is given by


$$\begin{array}{c} \tan\alpha_{x}=\frac{U_{x}}{U_{z}}=\frac{(\cos^{2}\phi +\kappa_{2}\sin^{2}\phi -\kappa_{1})\cos\theta \sin\theta }{(\cos^{2}\phi +\kappa_{2}\sin^{2}\phi )\cos^{2}\theta +\kappa_{1}\sin^{2}\theta } \end{array}$$ (39)

and the deflection angle $\alpha_{y}$ in the *y* direction is given by


$$\begin{array}{c} \tan\alpha_{y}=\frac{U_{y}}{U_{z}}=\frac{(\kappa_{2}-1)\cos\theta \cos\phi \sin\phi }{(\cos^{2}\phi +\kappa_{2}\sin^{2}\phi )\cos^{2}\theta +\kappa_{1}\sin^{2}\theta }, \end{array}$$ (40)

where $U_{x}$, $U_{y}$, and $U_{z}$ are given by Eq (14), and the quantities $\kappa_{1}$ and $\kappa_{2}$ are defined by $\kappa_{1}=U_{1}/U_{3}$ and $\kappa_{2}=U_{2}/U_{3}$. Referring to Eq (15) note that we can also define $\kappa_{1}=\zeta_{3}/\zeta_{1}$ and $\kappa_{2}=\zeta_{3}/\zeta_{2}$.

**Fig 7:**
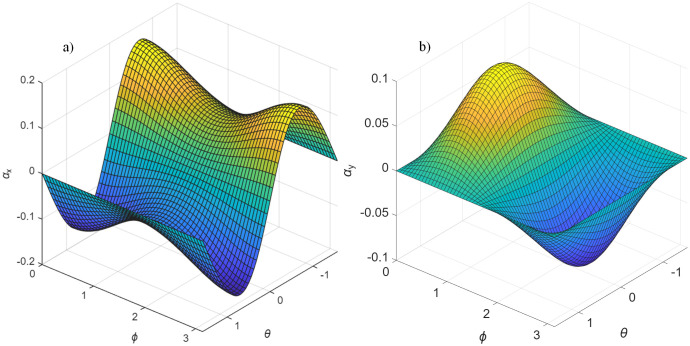
Surface plots of the deflection angles in the *x* and *y* directions. The deflection angles are given by Eqs (39) and (40), respectively, for $\kappa_{1}=1.4$ and $\kappa_{2}=1.2$.

Fig 7 shows surface plots of the deflection angles $\alpha_{x}$ and $\alpha_{y}$ in terms of θ and ϕ, both on the interval [0,π], for $\kappa_{1}=1.4$ and $\kappa_{2}=1.2$. These values of $\kappa_{1}=0.8$ and $\kappa_{2}$ are in the range of values relevant to the cuboid illustrated in Fig 1, for which $U_{1}>U_{2}>U_{3}$ and $\zeta_{1}<\zeta_{2}<\zeta_{3}$. It may be useful to look at the special cases of ϕ=0 and θ=0 which were considered in Eqs (21) and (22), respectively. Referring to Fig 7, we see that for ϕ=0, $\alpha_{x}$ is negative for 0<θ<π/2 and positive for π/2<θ<π, which corresponds to rotation about the $y^{\prime }$ axis; and for θ=0, $\alpha_{y}$ is positive for 0<ϕ<π/2 and negative for π/2<ϕ<π, which corresponds to rotation about the axis. This agrees with the discussion following Eqs (21) and (22).

#### 3.3.1. The drag coefficients along two axes are equal.

As in section 3.2.1, we now consider the special case $U_{3}=U_{2}$. Therefore, $\kappa_{2}=U_{2}/U_{3}=1$, and Eq (39) reduces to


$$\begin{array}{c} \tan\alpha_{x}=\frac{(1-\kappa_{1})\cos\theta \sin\theta }{\cos^{2}\theta +\kappa_{1}\sin^{2}\theta }, \end{array}$$ (41)

and Eq (40) reduces to $\tan\alpha_{y}=0$. Dividing the numerator and denominator by $\cos^{2}\theta$, Eq (41) may be written as


$$\begin{array}{c} \tan\alpha_{x}=\frac{(1-\kappa_{1})\tan\theta }{1+\kappa_{1}\tan^{2}\theta }. \end{array}$$ (42)

Eq (42) can be shown to be equivalent to Eq (36) of [2] for the case of an obliquely falling cylindrical rod with circular cross section, for which the angle θ is measured from the vertical, after making the transformation θ→θ+π/2. Here, we look at this equation in more detail, particularly extending the analysis for the domain of θ from [0,π/2] to [−π/2,π/2] and the domain of $\kappa_{1}$ from [1,2] to [2/3,2].

The upper limit on the domain for $\kappa_{1}$ is obtained by considering a long thin rod ($L_{3}\ll L_{1}$), which is known to have a value $\kappa_{1}=U_{1}/U_{3}=2$; that is, a long thin rod falls twice as fast in a direction parallel to its length as compared to the rod falling in a direction perpendicular to its length [2,23–25]. A long thin rod can be represented as a long thin prolate spheroid [32], so dividing Eq (26) by Eq (29), we also find $\kappa_{1}=U_{1}/U_{3}=2$.

The lower limit on the domain for $\kappa_{1}$ is obtained by considering a thin circular disk ($L_{1}\ll L_{3}$), which can be represented by a thin oblate spheroid [32]. So, dividing Eq (33) by Eq (36), we find $\kappa_{1}=U_{1}/U_{3}=2/3$; that is, the speed of a thin circular disk falling parallel to its symmetry axis is 2/3 the speed of the disk falling perpendicular to its symmetry axis.

Taking ∂/∂θ of Eq (36), we find that this function has two extrema as functions of $\kappa_{1}$ on the interval 0≤θ≤π, the coordinates of which are given by


$$\begin{array}{c} {\left(\theta ,\alpha_{x}\right)}_{\text{ext}}=\left(\tan^{-1}\frac{1}{\sqrt{\kappa_{1}}},\tan^{-1}\frac{1-\kappa_{1}}{2\sqrt{\kappa_{1}}}\right) \end{array}$$ (43)

and


$$\begin{array}{c} {\left(\theta ,\alpha_{x}\right)}_{\text{ext}}=\left(-\tan^{-1}\frac{1}{\sqrt{\kappa_{1}}},-\tan^{-1}\frac{1-\kappa_{1}}{2\sqrt{\kappa_{1}}}\right). \end{array}$$ (44)

**Fig 8:**
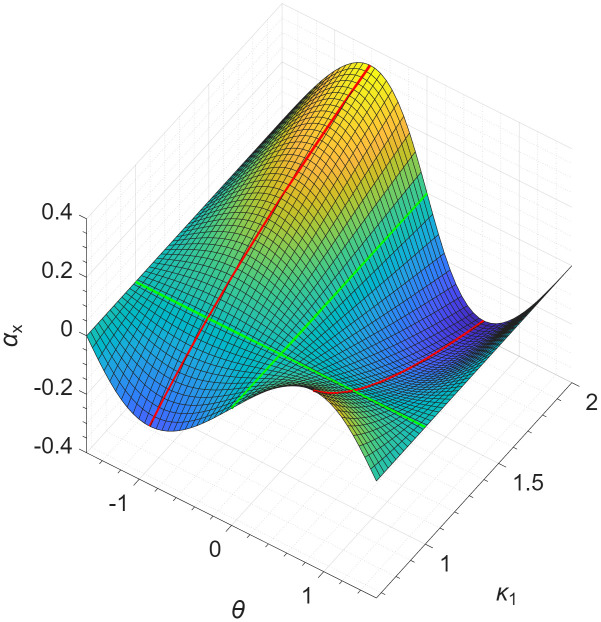
Surface plot of the deflection angle for the special case where the drag coefficients along two axes are equal. In this case $\kappa_{2}=1$, $\tan\alpha_{y}=0$, and the deflection angle $\alpha_{x}$ as a function of θ and $\kappa_{1}$ is given by Eq (36). The domain for θ is [−π/2,π/2] and the domain for $\kappa_{1}$ is [2/3,2]. The horizontal green lines divide the region into four quadrants, as described after Eq (44). The extrema are indicated by the red colored curves and determined by Eqs (43) and (44).

Fig 8 shows a surface plot of $\alpha_{x}$ as a function of θ and $\kappa_{1}$, as given by Eq (42). The horizontal green lines from the point $\left(\theta ,\kappa_{1},\alpha_{x})=(-\pi /2,1,0\right)$ to (π/2,1,0) and from the point $\left(\theta ,\kappa_{1},\alpha_{x})=(0,2/3,0\right)$ to (0,2,0) divide the region into four quadrants:

- Quadrant 1: $2/3\leq \kappa_{1}<1$ and 0<θ<π/2, for which $\alpha_{x}>0$.
- Quadrant 2: $2/3\leq \kappa_{1}<1$ and −π/2<θ<0, for which $\alpha_{x}<0$.
- Quadrant 3: $1<\kappa_{1}\leq 2$ and 0<θ<π/2, for which $\alpha_{x}<0$.
- Quadrant 4: $1<\kappa_{1}\leq 2$ and −π/2<θ<0, for which $\alpha_{x}>0$.

As noted previously, the interval $2/3<\kappa_{1}<2$ was determined by considering the limits of a thin disk for which $\kappa_{1}=U_{1}/U_{3}\to 2/3$ and a long thin rod for which $\kappa_{1}=U_{1}/U_{3}\to 2$. The extrema are indicated by the red colored curves and determined by Eqs (43) and (44).

### 3.4. Another application: Magnetic particles of various shapes in a gradient magnetic field

If instead of considering the motion of mass objects in a gravitational field, we consider the motion of magnetic objects resulting from a gradient magnetic field, the situation is significantly different. For example, consider the motion of a magnetic particle with rotational symmetry about its long axis, such as a prolate spheroid. In an external magnetic field, shape anisotropy by itself implies that the magnetic moment **m** of the spheroid will tend to align along the long axis of the spheroid. In addition, if there is a gradient in the magnetic field there will be an external force $F_{mag}$ acting on the spheroid given by


$$\begin{array}{c} F_{mag}=(m\cdot \nabla )B. \end{array}$$ (45)

If $F_{mag}$ is in the positive x direction and the magnetic field **B** makes an oblique angle θ with $F_{mag}$ then, in addition to the motion of the of the spheroid in the positive *x* direction, there will be a sideways component of velocity because of the interaction of the fluid with the spheroid. **An important point, distinguishing this from the gravitational field case, is that the angle between the long axis of the spheroid and the external force will be fixed, as long as the magnetic energy is much larger than the thermal energy.** Recall that for an object with size on the order of a micron or smaller in a gravitational field, the object will not maintain its orientation, but will randomly rotate due to thermal motion.

**Fig 9:**
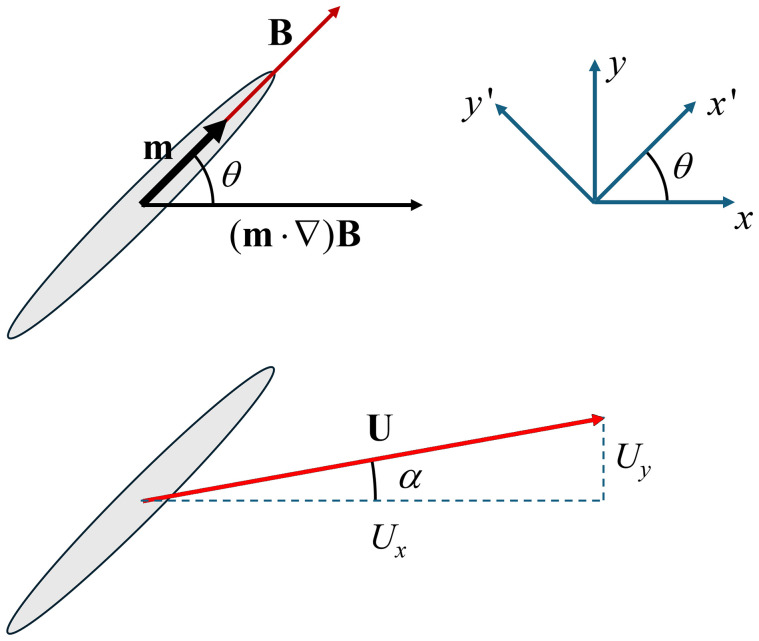
A prolate spheroidal magnetic particle in the presence of a gradient magnetic field. As the particle is drawn in the direction of the magnetic force, it is deflected to the side. For the angle between **B** and $F_{mag}$ we chose θ=45°. For the aspect ratio length/width we chose $L_{2}/L_{1}=10$. As derived in the text we found for the deflection angle, α=10.22°.

The field **B** and force $F_{mag}$ will lie in a plane and, without loss of generality we take this plane to be the *x*-*y* plane, since a coordinate system may always be chosen for which this is the case. Fig 9 shows the cross section of a prolate spheroidal magnetic particle in the presence of a gradient magnetic field. $F_{mag}$ is chosen to be along the *x* axis. The field **B**, and therefore the long axis of the particle, makes an angle of θ with $F_{mag}$.

Transformation from the laboratory frame to the body frame is provided by a rotation matrix as follows:


$$\begin{array}{c} \left(\begin{array}{c} x^{\prime }\\ y^{\prime } \end{array}\right)=𝐑(\theta )\left(\begin{array}{c} x\\ y \end{array}\right), \end{array}$$ (46)

where


$$\begin{array}{c} 𝐑(\theta )=\left(\begin{array}{cc} \cos\theta & \sin\theta \\ -\sin\theta & \cos\theta \end{array}\right). \end{array}$$ (47)

Transformation from the body frame back to the laboratory frame is given by:


$$\begin{array}{c} \left(\begin{array}{c} x\\ y \end{array}\right)=R^{T}(\theta )\left(\begin{array}{c} x^{\prime }\\ y^{\prime } \end{array}\right), \end{array}$$ (48)

where $R^{T}$ is the transpose (and inverse) of **R**.

Just as for the three-dimensional case, we first transform the force from the laboratory frame to the body frame:


$$\begin{array}{c} \left(\begin{array}{c} F_{mag,x^{\prime }}\\ F_{mag,y^{\prime }} \end{array}\right)=𝐑(\theta )\left(\begin{array}{c} F_{mag}\\ 0 \end{array}\right)=\left(\begin{array}{c} F_{mag}\cos\theta \\ -F_{mag}\sin\theta \end{array}\right). \end{array}$$ (49)

Since we are in the body frame, the mobility matrix is diagonal, and Eq (1) gives


$$\begin{array}{c} \left(\begin{array}{c} U_{x^{\prime }}\\ U_{y^{\prime }} \end{array}\right)=\left(\begin{array}{cc} \mu_{1} & 0\\ 0 & \mu_{2} \end{array}\right)\left(\begin{array}{c} F_{mag}\cos\theta \\ -F_{mag}\sin\theta \end{array}\right)=\left(\begin{array}{c} \mu_{1}F_{mag}\cos\theta \\ -\mu_{2}F_{mag}\sin\theta \end{array}\right). \end{array}$$ (50)

We now transform back to the laboratory frame


$$\begin{array}{c} \left(\begin{array}{c} U_{x}\\ U_{y} \end{array}\right)=R^{T}(\theta )\left(\begin{array}{c} \mu_{1}F_{mag}\cos\theta \\ -\mu_{2}F_{mag}\sin\theta \end{array}\right)=F_{mag}\left(\begin{array}{c} (1/\zeta_{1})\cos^{2}\theta +(1/\zeta_{2})\sin^{2}\theta \\ (1/\zeta_{1}-1/\zeta_{2})\cos\theta \sin\theta \end{array}\right), \end{array}$$ (51)

where the diagonal mobility matrix values have been rewritten in terms of the drag coefficients via the friction matrix Eq (2), that is, $\mu_{1}=1/\zeta_{1}$ and $\mu_{2}=1/\zeta_{2}$ was used. Here $\zeta_{1}$ is the drag coefficient parallel to the long axis and $\zeta_{2}$ is the drag coefficient perpendicular to the long axis. From Eq (51) we find for the ratio $U_{y}/U_{x}$ and for the deflection angle α


$$\begin{array}{c} \tan\alpha =\frac{U_{y}}{U_{x}}=\frac{(1-\zeta_{1}/\zeta_{2})\cos\theta \sin\theta }{\cos^{2}\theta +(\zeta_{1}/\zeta_{2})\sin^{2}\theta }. \end{array}$$ (52)

Eqs (51) and (52) will apply to any magnetic particle with rotational symmetry about its long axis, such as a cylindrical rod or a prolate spheroid as illustrated in Fig 9. In fact, it will apply to any of the shapes seen in Fig 4a-4e. However, it is not necessary that the particle has three mutually perpendicular planes of symmetry to a high degree of tolerance to maintain its orientation. For the object will maintain the angle θ between the magnetic field and the magnetic force *even though the object deviates significantly from having three mutually perpendicular planes of symmetry*, as long as the magnetic moment and magnetic field are sufficiently large.

To determine the deflection angle α from Eq (52), we need to determine the ratio of the drag coefficients parallel to and perpendicular to the long axis. In general, these would need to be determined numerically, experimentally, or analytically (for a sufficiently simple geometry). From the exact analytic solutions Eqs (24) and (27), recalling that ζ=F/U, and defining $A=\ln((1+e_{p})/(1-e_{p}))$ we find for a prolate spheroid


$$\begin{array}{c} \frac{\zeta_{1}}{\zeta_{2}}=\frac{1}{2}\frac{A(3e_{p}^{2}-1)+2e_{p}}{A(1+e_{p}^{2})-2e_{p}}, \end{array}$$ (53)

where $e_{p}=\sqrt{1-{L_{2}}^{2}/{L_{1}}^{2}}$ is the eccentricity of the spheroid. Inserting θ=45° and $L_{2}/L_{1}=1/10$ into Eq (53), we find $\zeta_{1}/\zeta_{2}=0.6945$. Inserting this value into Eq (52), we find the ratio $U_{y}/U_{x}=0.1803$ and the deflection angle α=10.22°, as illustrated in Fig 9.

In summary, Eq (52) gives the deflection angle in terms of the orientation angle for an elongated magnetic particle with roughly three mutually perpendicular planes of symmetry for which the magnetic moment is parallel to the long axis of the particle and for which the two drag coefficients associated with flow perpendicular to the long axis are roughly equal. For example, as noted previously, it will apply to any of the shapes illustrated in Fig 4a-4e, and also apply to rods with a roughly regular polygonal cross section, as discussed at the end of section 3.2.2. Unless one has a sufficiently simple geometry, as we had for a prolate spheroid outlined above, the drag coefficients or their ratio will need to be determined numerically or experimentally.

#### 3.4.1. The three-dimensional case.

**Fig 10:**
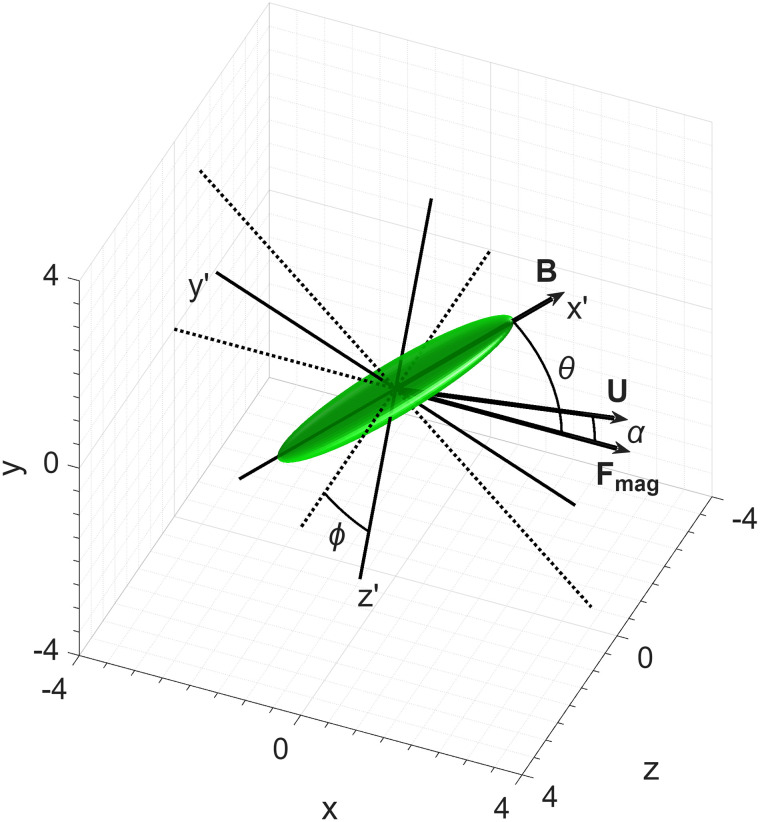
An elongated triaxial ellipsoidal magnetic particle in the presence of a gradient magnetic field. Triaxial implies that the lengths of all the minor axes are different from one another, that is a≠b≠c and a≠c. Therefore, the two drag coefficients associated with flow perpendicular to the long axis of a particle are different from one another. Because of thermal motion, the particle rotates randomly about the magnetic field direction. As the particle is drawn in the direction of the magnetic force, it is deflected to the side. As derived in the text for a=50nm, b=5nm, c=10nm, and θ=45° we found α=9.057°.

A question that may arise is what would happen if the two drag coefficients associated with flow perpendicular to the long axis of a particle are significantly different from one another. Although the long axis of the particle is constrained to be parallel to the magnetic field, there would be no such constraint on the orientation of the particle about the magnetic field direction. Assuming the particle is sufficiently small, we would expect that the particle would randomly rotate about the magnetic field direction because of thermal motion. To derive the motion of such a particle, we add a dimension *z* perpendicular to the *x*-*y* plane as illustrated in Fig 10.

Transformation from the laboratory frame to the body frame is provided by rotation matrices as follows (details of the following may be found in the PDF S2 File Euler_phi_average.pdf of the Matlab live script in the supporting information.):


$$\begin{array}{c} \left(\begin{array}{c} x^{\prime }\\ y^{\prime }\\ z^{\prime } \end{array}\right)=R_{x^{\prime }}(\phi )R_{z^{\prime }}(\theta )\left(\begin{array}{c} x\\ y\\ z \end{array}\right), \end{array}$$ (54)

where


$$\begin{array}{c} R_{x^{\prime }}(\phi )=\left(\begin{array}{ccc} 1 & 0 & 0\\ 0 & \cos\phi & \sin\phi \\ 0 & -\sin\theta & \cos\theta \end{array}\right) \end{array}$$ (55)

and


$$\begin{array}{c} R_{z^{\prime }}(\phi )=\left(\begin{array}{ccc} \cos\theta & \sin\theta & 0\\ -\sin\theta & \cos\theta & 0\\ 0 & 0 & 1 \end{array}\right). \end{array}$$ (56)

Transformation from the body frame back to the laboratory frame is given by:


$$\begin{array}{c} \left(\begin{array}{c} x\\ y\\ z \end{array}\right)=R_{z^{\prime }}^{T}(\theta )R_{x^{\prime }}^{T}(\phi )\left(\begin{array}{c} x^{\prime }\\ y^{\prime }\\ z^{\prime } \end{array}\right), \end{array}$$ (57)

where the superscript refers to the transpose (and inverse) of the matrices given in Eqs (55) and (56).

We first transform the force from the laboratory frame to the body frame:


$$\begin{array}{c} \left(\begin{array}{c} F_{mag,x^{\prime }}\\ F_{mag,y^{\prime }}\\ F_{mag,z^{\prime }} \end{array}\right)=R_{x^{\prime }}(\phi )R_{z^{\prime }}(\theta )\left(\begin{array}{c} F_{mag}\\ 0\\ 0 \end{array}\right)=\left(\begin{array}{c} F_{mag}\cos\theta \\ -F_{mag}\sin\theta \cos\phi \\ F_{mag}\sin\theta \sin\phi \end{array}\right). \end{array}$$ (58)

Since we are in the body frame, the mobility matrix is diagonal, and Eq (1) gives


$$\begin{array}{c} \left(\begin{array}{c} U_{x^{\prime }}\\ U_{y^{\prime }}\\ U_{z^{\prime }} \end{array}\right)=\left(\begin{array}{ccc} \mu_{1} & 0 & 0\\ 0 & \mu_{2} & 0\\ 0 & 0 & \mu_{3} \end{array}\right)\left(\begin{array}{c} F_{mag}\cos\theta \\ -F_{mag}\sin\theta \cos\phi \\ F_{mag}\sin\theta \sin\phi \end{array}\right)=\left(\begin{array}{c} \mu_{1}F_{mag}\cos\theta \\ -\mu_{2}F_{mag}\sin\theta \cos\phi \\ \mu_{3}F_{mag}\sin\theta \sin\phi \end{array}\right). \end{array}$$ (59)

We now transform back to the laboratory frame


$$\begin{array}{cc} \left(\begin{array}{c} U_{x}\\ U_{y}\\ U_{z} \end{array}\right) & =R_{z^{\prime }}^{T}(\theta )R_{x^{\prime }}^{T}(\phi )\left(\begin{array}{c} \mu_{1}F_{mag}\cos\theta \\ -\mu_{2}F_{mag}\sin\theta \cos\phi \\ \mu_{3}F_{mag}\sin\theta \sin\phi \end{array}\right)\\ & =F_{mag}\left(\begin{array}{c} \mu_{1}\cos^{2}\theta +\left(\mu_{2}\cos^{2}\phi +\mu_{3}\sin^{2}\phi \right)\sin^{2}\theta \\ \left(\mu_{1}-\mu_{2}\cos^{2}\phi -\mu_{3}\sin^{2}\phi \right)\cos\theta \sin\theta \\ \left(\mu_{3}-\mu_{2}\right)\cos\phi \sin\phi \sin\theta \end{array}\right), \end{array}$$ (60)

where $\mu_{1}=1/\zeta_{1}$, $\mu_{2}=1/\zeta_{2}$, and $\mu_{3}=1/\zeta_{3}$.

Assuming the particle is small enough, we expect it to randomly rotate about the magnetic field direction due to thermal motion. Therefore, it is necessary to average over ϕ as


$$\begin{array}{c} U_{avg}(\theta )=\frac{\int d\phi \;U(\phi ,\theta )}{\int d\phi }=\frac{1}{2\pi }\int d\phi \;U(\phi ,\theta ). \end{array}$$ (61)

Performing the integral we find


$$\begin{array}{cc} U_{avg}(\theta ) & =F_{mag}\left(\begin{array}{c} \mu_{1}\cos^{2}\theta +\frac{\mu_{2}+\mu_{3}}{2}\sin^{2}\theta \\ \left(\mu_{1}-\frac{\mu_{2}+\mu_{3}}{2}\right)\cos\theta \sin\theta \\ 0 \end{array}\right)=F_{mag}\left(\begin{array}{c} \mu_{1}\cos^{2}\theta +{\bar{\mu }}_{⟂}\sin^{2}\theta \\ (\mu_{1}-{\bar{\mu }}_{⟂})\cos\theta \sin\theta \\ 0 \end{array}\right)\\ & =F_{mag}\left(\begin{array}{c} (1/\zeta_{1})\cos^{2}\theta +(1/{\bar{\zeta }}_{⟂})\sin^{2}\theta \\ (1/\zeta_{1}-1/{\bar{\zeta }}_{⟂})\cos\theta \sin\theta \\ 0 \end{array}\right), \end{array}$$ (62)

where ${\bar{\zeta }}_{⟂}$ is the average drag coefficient perpendicular to the long axis of the particle, determined by $1/{\bar{\zeta }}_{⟂}=(1/2)\;(1/\zeta_{2}+1/\zeta_{3})$. From Eq (62) we find for the ratio $U_{y}/U_{x}$ and for the deflection angle α


$$\begin{array}{c} \tan\alpha =\frac{U_{y}}{U_{x}}=\frac{(1-\zeta_{1}/{\bar{\zeta }}_{⟂})\cos\theta \sin\theta }{\cos^{2}\theta +(\zeta_{1}/{\bar{\zeta }}_{⟂})\sin^{2}\theta }. \end{array}$$ (63)

Eq (63) gives the deflection angle in terms of the orientation angle for an elongated magnetic particle with roughly three mutually perpendicular planes of symmetry for which the magnetic moment is parallel to the long axis of the particle, the particle is small enough so that it rotates randomly about the magnetic field direction because of thermal motion, and the two drag coefficients associated with flow perpendicular to the long axis are in general *not equal* to one another. If these two drag coefficients are equal, then Eq (63) reduces to Eq (52).

To calculate the deflection angle for the ellipsoid illustrated in Fig 10, we need the drag coefficient along each principal axis. Although the drag coefficients for a general ellipsoid cannot be written in terms of elementary functions as was the case for a prolate spheroid or an oblate spheroid given by Eqs (24) and (31), respectively, they can be written in terms of integrals. The drag coefficients for a general ellipsoid are given by [34,35]


$$\begin{array}{c} \zeta_{i}=6\pi \eta R_{i}\;\;\;\;\;\;\;\;\;\;(i=1,2,3), \end{array}$$ (64)

where


$$\begin{array}{c} R_{i}=\frac{8}{3}\frac{1}{\chi_{0}+\alpha_{0}a_{i}^{2}}\;\;\;\;\;\;\;\;\;\;(i=1,2,3), \end{array}$$ (65)

$$\begin{array}{c} \chi_{0}=\int_{0}^{\infty }\frac{d\lambda }{\Delta }, \end{array}$$ (66)

$$\begin{array}{c} \alpha_{i}=\int_{0}^{\infty }\frac{d\lambda }{(a_{i}^{2}+\lambda )\Delta }\;\;\;\;\;\;\;\;\;\;(i=1,2,3), \end{array}$$ (67)

$$\begin{array}{c} \Delta =\sqrt{\left(a^{2}+\lambda )(b^{2}+\lambda )(c^{2}+\lambda \right)}, \end{array}$$ (68)

where $a_{1}=a=L_{1}/2$, $a_{2}=b=L_{2}/2$, and $a_{3}=c=L_{3}/2$ are the minor axes of the ellipsoid along the $x^{\prime }$, $y^{\prime }$, and $z^{\prime }$ directions, respectively. Using Matlab (see S3 File ellipsoid_drag.pdf in the supporting information) with a=50nm, b=5nm, c=10nm, η=1.00mPa·s, and θ=45° we find $\zeta_{1}=2.943\times 10^{-10}\;kg/s$, $\zeta_{2}=4.274\times 10^{-10}\;kg/s$, $\zeta_{3}=3.864\times 10^{-10}\;kg/s$, ${\bar{\zeta }}_{⟂}=4.059\times 10^{-10}\;kg/s$, $\zeta_{1}/{\bar{\zeta }}_{⟂}=0.7250$, $U_{y}/U_{x}=0.1594$, and α=9.057°.

#### 3.4.2. Relaxation time about the long axis of the ellipsoid.

As pointed out in the last section, although the long axis of the ellipsoid aligns with the magnetic field, the particle is still free to rotate about its long axis. The rotational diffusion coefficient about the $i^{th}$ axis is given by [36]


$$\begin{array}{c} D_{ri}=\frac{k_{B}T}{\zeta_{ri}}\;\;\;\;\;\;\;\;\;\;(i=1,2,3) \end{array}$$ (69)

where the rotational drag coefficients are given by


$$\begin{array}{c} \zeta_{r1}=\frac{16\pi \eta }{3}\frac{b^{2}+c^{2}}{b^{2}\beta +c^{2}\gamma },\;\;\;\;\zeta_{r2}=\frac{16\pi \eta }{3}\frac{a^{2}+c^{2}}{a^{2}\alpha +c^{2}\gamma },\;\;\;\;\zeta_{r3}=\frac{16\pi \eta }{3}\frac{a^{2}+b^{2}}{a^{2}\alpha +b^{2}\beta }, \end{array}$$ (70)

and


$$\begin{array}{c} \alpha =\int_{0}^{\infty }\frac{d\lambda }{(a^{2}+\lambda )\Delta },\;\;\;\;\beta =\int_{0}^{\infty }\frac{d\lambda }{(b^{2}+\lambda )\Delta },\;\;\;\;\gamma =\int_{0}^{\infty }\frac{d\lambda }{(c^{2}+\lambda )\Delta }, \end{array}$$ (71)

$$\begin{array}{c} \Delta =\sqrt{\left(a^{2}+\lambda )(b^{2}+\lambda )(c^{2}+\lambda \right)}, \end{array}$$ (72)

where *a*, *b*, and *c* are the minor axes of the ellipsoid, and $k_{B}$ is Boltzmann’s constant.

Since the magnetic field constrains the ellipsoid to rotate only about its long axis as illustrated in Fig 10, we only calculate $D_{r1}$ from Eq (69). Also, because there is only rotation about the long axis we have for the relaxation time about the long axis [37]


$$\begin{array}{c} \tau_{1}=\frac{1}{2D_{r1}}=\frac{\zeta_{r1}}{2k_{B}T}. \end{array}$$ (73)

Note the factor of two in the denominator. If the ellipsoid were instead allowed to rotate freely in three-dimensional space, this factor would instead be 6. The relaxation time is the time for the particle to rotate about its long axis by 1 radian [38]. From this we obtain the characteristic frequency


$$\begin{array}{c} f_{1}=\frac{1}{2\pi \tau_{1}}=\frac{k_{B}T}{\pi \zeta_{r1}}. \end{array}$$ (74)

For the ellipsoid in the last section, that has a length $L_{1}=100\;nm$ and widths of $L_{2}=10\;nm$ and $L_{3}=20\;nm$ (suspended in water at 20° C) we obtain the rotational drag coefficient $\zeta_{r1}=5.450\times 10^{-26}\;J\cdot s$, the relaxation time $\tau_{1}=6.735\;\mu s$, and the characteristic frequency $f_{1}=23.63\;kHz$. Details may be found in the Matlab program S4 File ellipsoid_drag_rot.pdf in the supporting information.

## 4. Conclusions

In this paper we first derived a general formula for the three components of velocity for an object of arbitrary orientation and uniform density and with three mutually perpendicular planes of symmetry falling through a viscous fluid in the Stokes flow limit. This is given in terms of the speeds of the object falling along each of its three mutually principal axes or in terms of the effective weight and drag coefficients along each of its principal axes. The orientation of the object is specified using Euler angles. In addition to the downward component of velocity, the object in general will have sideways components of velocity.

The speeds along each of the principal axes, for example, could be measured experimentally by releasing the object in the fluid parallel to each of its three principal axes. These three speeds could also be calculated numerically or analytically for a sufficiently simple geometry. Similarly, the drag coefficients could be measured along each of its principal axes either experimentally, numerically, or analytically for a sufficiently simple geometry. It is assumed that the object is far from boundaries to minimize any interaction between the object and boundary. Once these three parameters are measured, the velocity of the object, including sideways components, will be determined for an arbitrary orientation. Also, equations were derived for the deflection angles in the *x* and *y* directions, and surface plots were made for the deflection angles.

We also derived equations for the motion of magnetic particles with elongated shapes, such as magnetic nanorods and magnetic nanoellipsoids, in a gradient magnetic field. If the magnetic field makes an oblique angle with the magnetic force, the particles will not move directly in the direction of the magnetic force but will also have a sideways motion relative to the magnetic force. An important distinction between a magnetic object subjected to a magnetic force compared to an object subjected to a gravitational field, is that the orientation of the object in the magnetic force case is determined by the angle between the magnetic force and magnetic field. Therefore, to change the angle of the object relative to the magnetic force, it is necessary to change the angle between the magnetic field and the magnetic force. In contrast, for an object in a gravitational field, it is only necessary to physically change the orientation of the object and then release it in the fluid.

This implies that for the gravitational case, the object must have three perpendicular planes of symmetry to a high degree of tolerance, in order for the object to maintain its orientation as it falls. Otherwise it may rotate. On the other hand, for the magnetic force case, since the orientation of the object relative to the magnetic force is simply determined by the angle between the magnetic field and the magnetic force, the symmetry of the object can deviate significantly from having three mutually perpendicular planes of symmetry.

Another important difference between the gravitational and magnetic cases is that for the gravitational case, if the object is on the order of a micron or smaller in size, the object will constantly rotate as it falls through the fluid because of thermal motion. In contrast, for the magnetic case, the object will maintain its orientation with respect to the magnetic force even if it is on the order of a micron or smaller in size, as long as the magnetic energy is much greater than the thermal energy.

In this paper the question was asked as to what would happen if the two drag coefficients associated with motion perpendicular to the long axis of an elongated magnetic particle are *not equal*. In this case for a particle on the order of a micron or smaller in size we expect that the particle would randomly rotate about the magnetic field vector due to thermal motion. We derived an equation for the deflection angle, as well as an equation giving the characteristic frequency of this random rotation. Because of this rotation the drag coefficient perpendicular to the long axis of the particle is an average given by $1/{\bar{\zeta }}_{⟂}=(1/2)(1/\zeta_{2}+1/\zeta_{3})$. It would be interesting to test this prediction.

As noted in the reviews and other references cited in the Introduction, applications of our magnetic analysis of the motion of micro-sized and smaller objects in a viscous fluid in the Stokes limit include drug targeting and cell studies. The optimization of drug delivery also depends on the shapes of the drug carrier vehicles. The orientation of the objects relative to the driving force determines the motion direction and thus is of particular importance to understanding and making the particle fluid migration more efficient. The flow of magnetic particles may be better understood to the extent that they are approximated by the shapes studied in this paper.

## Supporting information

S1 File Euler_average.pdf. PDF file containing the Matlab live script used in section 3.1.1.
(PDF)

S2 File Euler_phi_average.pdf. PDF file containing the Matlab live script used in section 3.4.1.
(PDF)

S3 File ellipsoid_drag.pdf. PDF file containing the Matlab live script used in section 3.4.1.
(PDF)

S4 File ellipsoid_drag_rot.pdf. PDF file containing the Matlab live script used in section 3.4.2.
(PDF)
