# Supplementary material for: Objects with three orthogonal symmetry planes: Oblique driving forces and Stokes flow motion
Source: PLoS One. 2026 Jul 6;21(7):e0352508. doi: 10.1371/journal.pone.0352508 (PMC13336483; doi:10.1371/journal.pone.0352508)
Supplement: S1 File — PDF file containing the Matlab live script used in section 3.1.1. (PDF) [file pone.0352508.s001.pdf]

```
clearvars;
syms phi theta psi W_eff mu_1 mu_2 mu_3

% transpose of Eq (4) from paper
RT_phi = [1,0,0; 0, cos(phi), -sin(phi); 0, sin(phi), cos(phi)]
```

RT_phi =

$$\begin{pmatrix} 1 & 0 & 0 \\ 0 & \cos(\phi) & -\sin(\phi) \\ 0 & \sin(\phi) & \cos(\phi) \end{pmatrix}$$

```
% transpose of Eq (5)
RT_theta = [cos(theta), 0, sin(theta); 0,1,0;-sin(theta),0,cos(theta)]
```

RT_theta =

$$\begin{pmatrix} \cos(\theta) & 0 & \sin(\theta) \\ 0 & 1 & 0 \\ -\sin(\theta) & 0 & \cos(\theta) \end{pmatrix}$$

```
% transpose of Eq (6)
RT_psi = [cos(psi), -sin(psi), 0; sin(psi), cos(psi), 0; 0,0,1]
```

RT_psi =

$$\begin{pmatrix} \cos(\psi) & -\sin(\psi) & 0 \\ \sin(\psi) & \cos(\psi) & 0 \\ 0 & 0 & 1 \end{pmatrix}$$

```
% Total rotation matrix from body frame to lab frame Eq (7)
RT = RT_psi * RT_theta * RT_phi
```

RT =

$$\begin{pmatrix} \cos(\psi)\,\cos(\theta) & \cos(\psi)\,\sin(\phi)\,\sin(\theta) - \cos(\phi)\,\sin(\psi) & \sin(\phi)\,\sin(\psi) + \cos(\phi)\,\cos(\psi)\,\sin(\theta) \\ \cos(\theta)\,\sin(\psi) & \cos(\phi)\,\cos(\psi) + \sin(\phi)\,\sin(\psi)\,\sin(\theta) & \cos(\phi)\,\sin(\psi)\,\sin(\theta) - \cos(\psi)\,\sin(\phi) \\ -\sin(\theta) & \cos(\theta)\,\sin(\phi) & \cos(\phi)\,\cos(\theta) \end{pmatrix}$$

```
% Velocity in body frame Eq (9)
U_prime = W_eff * [-mu_1*sin(theta); mu_2*cos(theta)*sin(phi);
mu_3*cos(theta)*cos(phi)]
```

U_prime =

$$\begin{pmatrix} -W_{\mathrm{eff}}\,\mu_1\,\sin(\theta) \\ W_{\mathrm{eff}}\,\mu_2\,\cos(\theta)\,\sin(\phi) \\ W_{\mathrm{eff}}\,\mu_3\,\cos(\phi)\,\cos(\theta) \end{pmatrix}$$

```
% Veocity in lab frame Eq (18)
U = simplify(RT * U_prime)
```

U =

$$\begin{pmatrix} W_{\text{eff}}\,\mu_3\,\cos(\phi)\,\cos(\theta)\,(\sin(\phi)\,\sin(\psi)+\cos(\phi)\,\cos(\psi)\,\sin(\theta)) - W_{\text{eff}}\,\mu_2\,\cos(\theta)\,\sin(\phi)\,(\cos(\phi)\,\sin(\psi) \text{[illegible]} \\ W_{\text{eff}}\,\mu_2\,\cos(\theta)\,\sin(\phi)\,(\cos(\phi)\,\cos(\psi)+\sin(\phi)\,\sin(\psi)\,\sin(\theta)) - W_{\text{eff}}\,\mu_3\,\cos(\phi)\,\cos(\theta)\,(\cos(\psi)\,\sin(\phi) \text{[illegible]} \\ W_{\text{eff}}\left(\mu_3\cos(\phi)^2\cos(\theta)^2+\mu_2\cos(\theta)^2\sin(\phi)^2+\mu_1\,\text{sin[illegible]}\right. \end{pmatrix}$$

```
Us = subs(U, psi, 0)
```

Us =

$$\begin{pmatrix} W_{\text{eff}}\,\mu_3\,\cos(\theta)\,\sin(\theta)\cos(\phi)^2 + W_{\text{eff}}\,\mu_2\,\cos(\theta)\,\sin(\theta)\sin(\phi)^2 - W_{\text{eff}}\,\mu_1\,\cos(\theta)\,\sin(\theta) \\ W_{\text{eff}}\,\mu_2\,\cos(\phi)\,\cos(\theta)\,\sin(\phi) - W_{\text{eff}}\,\mu_3\,\cos(\phi)\,\cos(\theta)\,\sin(\phi) \\ W_{\text{eff}}\left(\mu_3\cos(\phi)^2\cos(\theta)^2+\mu_2\cos(\theta)^2\sin(\phi)^2+\mu_1\sin(\theta)^2\right) \end{pmatrix}$$

```
% Integrate U over psi from 0 to 2*pi
U_int = int(U, psi, 0, 2*pi)
```

U_int =

$$\begin{pmatrix} 0 \\ 0 \\ -2\,\pi\,W_{\text{eff}}\left(\mu_1\left(\cos(\theta)^2-1\right)-\mu_3\cos(\phi)^2\cos(\theta)^2+\mu_2\cos(\theta)^2\left(\cos(\phi)^2-1\right)\right) \end{pmatrix}$$

```
% Now integrate over phi fron 0 to 2*pi
U_int = int(U_int, phi, 0, 2*pi)
```

U_int =

$$\begin{pmatrix} 0 \\ 0 \\ 2\,W_{\text{eff}}\,\pi^2\left(2\,\mu_1-2\,\mu_1\cos(\theta)^2+\mu_2\cos(\theta)^2+\mu_3\cos(\theta)^2\right) \end{pmatrix}$$

```
% Now integrate over theta weighted by cos(theta) from -pi/2 to pi/2
U_int = int(cos(theta)*U_int, theta, -pi/2, pi/2)
```

U_int =

$$\begin{pmatrix} 0 \\ 0 \\ \dfrac{8\,W_{\text{eff}}\,\pi^2\,(\mu_1+\mu_2+\mu_3)}{3} \end{pmatrix}$$

```
% Calculate normilazation coefficient
norm = int(1, psi, 0, 2*pi)
```

norm = $2\,\pi$

```
norm = int(norm, phi, 0, 2*pi)
```

norm = $4\,\pi^2$

```
norm = int(cos(theta)*norm, theta, -pi/2, pi/2)
```

norm = $8\pi^2$

```
% This gives the average velocity over all Euler angles Eq (20)
U_avg = U_int/norm
```

U_avg =

$$\begin{pmatrix} 0 \\ 0 \\ \frac{W_{\mathrm{eff}}\,(\mu_1 + \mu_2 + \mu_3)}{3} \end{pmatrix}$$
