# Supplementary material for: Objects with three orthogonal symmetry planes: Oblique driving forces and Stokes flow motion
Source: PLoS One. 2026 Jul 6;21(7):e0352508. doi: 10.1371/journal.pone.0352508 (PMC13336483; doi:10.1371/journal.pone.0352508)
Supplement: S2 File — PDF file containing the Matlab live script used in section 3.4.1. (PDF) [file pone.0352508.s002.pdf]

```
clearvars;
syms phi theta F_mag mu_1 mu_2 mu_3

R_phi = [1,0,0; 0, cos(phi), sin(phi); 0, -sin(phi), cos(phi)]
```

R_phi =

$$\begin{pmatrix} 1 & 0 & 0 \\ 0 & \cos(\phi) & \sin(\phi) \\ 0 & -\sin(\phi) & \cos(\phi) \end{pmatrix}$$

```
R_theta = [cos(theta), sin(theta), 0; -sin(theta), cos(theta), 0; 0,0,1]
```

R_theta =

$$\begin{pmatrix} \cos(\theta) & \sin(\theta) & 0 \\ -\sin(\theta) & \cos(\theta) & 0 \\ 0 & 0 & 1 \end{pmatrix}$$

```
RT_phi = transpose(R_phi)
```

RT_phi =

$$\begin{pmatrix} 1 & 0 & 0 \\ 0 & \cos(\phi) & -\sin(\phi) \\ 0 & \sin(\phi) & \cos(\phi) \end{pmatrix}$$

```
RT_theta = transpose(R_theta)
```

RT_theta =

$$\begin{pmatrix} \cos(\theta) & -\sin(\theta) & 0 \\ \sin(\theta) & \cos(\theta) & 0 \\ 0 & 0 & 1 \end{pmatrix}$$

```

RT = RT_theta * RT_phi
```

RT =

$$\begin{pmatrix} \cos(\theta) & -\cos(\phi)\sin(\theta) & \sin(\phi)\sin(\theta) \\ \sin(\theta) & \cos(\phi)\cos(\theta) & -\cos(\theta)\sin(\phi) \\ 0 & \sin(\phi) & \cos(\phi) \end{pmatrix}$$

```
F_prime = R_phi * R_theta * [F_mag;0;0]
```

F_prime =

$$\begin{pmatrix} F_{\mathrm{mag}}\cos(\theta) \\ -F_{\mathrm{mag}}\cos(\phi)\sin(\theta) \\ F_{\mathrm{mag}}\sin(\phi)\sin(\theta) \end{pmatrix}$$

```

U_prime = [mu_1,0,0;0,mu_2,0;0,0,mu_3] * F_prime
```

U_prime =

$$\begin{pmatrix} F_{mag}\,\mu_1\cos(\theta) \\ -F_{mag}\,\mu_2\cos(\phi)\sin(\theta) \\ F_{mag}\,\mu_3\sin(\phi)\sin(\theta) \end{pmatrix}$$

```
U = RT * U_prime
```

U =

$$\begin{pmatrix} F_{mag}\,\mu_2\cos(\phi)^2\sin(\theta)^2 + F_{mag}\,\mu_1\cos(\theta)^2 + F_{mag}\,\mu_3\sin(\phi)^2\sin(\theta)^2 \\ -F_{mag}\,\mu_2\cos(\theta)\sin(\theta)\cos(\phi)^2 - F_{mag}\,\mu_3\cos(\theta)\sin(\theta)\sin(\phi)^2 + F_{mag}\,\mu_1\cos(\theta)\sin(\theta) \\ F_{mag}\,\mu_3\cos(\phi)\sin(\phi)\sin(\theta) - F_{mag}\,\mu_2\cos(\phi)\sin(\phi)\sin(\theta) \end{pmatrix}$$

```
% Integrate U over phi fron 0 to 2*pi
U_int = int(U, phi, 0, 2*pi)
```

U_int =

$$\begin{pmatrix} \pi\,F_{mag}\,\left(2\,\mu_1 - 2\,\mu_1\sin(\theta)^2 + \mu_2\sin(\theta)^2 + \mu_3\sin(\theta)^2\right) \\ -\dfrac{\pi\,F_{mag}\sin(2\,\theta)\,(\mu_2 - 2\,\mu_1 + \mu_3)}{2} \\ 0 \end{pmatrix}$$

```
% Calculate normilazation coefficient
norm = int(1, phi, 0, 2*pi)
```

norm = $2\,\pi$

```
U_avg = U_int/norm
```

U_avg =

$$\begin{pmatrix} \dfrac{F_{mag}\,\left(2\,\mu_1 - 2\,\mu_1\sin(\theta)^2 + \mu_2\sin(\theta)^2 + \mu_3\sin(\theta)^2\right)}{2} \\ -\dfrac{F_{mag}\sin(2\,\theta)\,(\mu_2 - 2\,\mu_1 + \mu_3)}{4} \\ 0 \end{pmatrix}$$
