# Supplementary material for: Objects with three orthogonal symmetry planes: Oblique driving forces and Stokes flow motion
Source: PLoS One. 2026 Jul 6;21(7):e0352508. doi: 10.1371/journal.pone.0352508 (PMC13336483; doi:10.1371/journal.pone.0352508)
Supplement: S3 File — PDF file containing the Matlab live script used in section 3.4.1. (PDF) [file pone.0352508.s003.pdf]

```
clearvars
theta = pi/4;
eta = 1; % dynamic viscosity
% minor axes of ellipsoid
a = 5;
b = 0.5;
c = 1;
f = @(x) a*b*c./sqrt((a^2+x).*(b^2+x).*(c^2+x));
q = integral(f, 0, inf);

fa = @(x) a*b*c./((a^2+x).*sqrt((a^2+x).*(b^2+x).*(c^2+x)));
qa = integral(fa, 0, inf);

fb = @(x) a*b*c./((b^2+x).*sqrt((a^2+x).*(b^2+x).*(c^2+x)));
qb = integral(fb, 0, inf);

fc = @(x) a*b*c./((c^2+x).*sqrt((a^2+x).*(b^2+x).*(c^2+x)));
qc = integral(fc, 0, inf);

Ra = (8/3)*a*b*c/(q + qa*a^2);
Rb = (8/3)*a*b*c/(q + qb*b^2);
Rc = (8/3)*a*b*c/(q + qc*c^2);

zeta_1 = 6*pi*eta*Ra
```

```
zeta_1 =
29.4265
```

```
zeta_2 = 6*pi*eta*Rb
```

```
zeta_2 =
42.7432
```

```
zeta_3 = 6*pi*eta*Rc
```

```
zeta_3 =
38.6387
```

```
zeta_perp = 2/(1/zeta_2 + 1/zeta_3)
```

```
zeta_perp =
40.5875
```

```
zeta_1d2 = zeta_1/zeta_perp
```

```
zeta_1d2 =
0.7250
```

```
UydUx = (1-zeta_1d2)*cos(theta)*sin(theta)/(cos(theta)^2 + zeta_1d2*sin(theta)^2)
```

```
UydUx =
0.1594
```

```
% deflection angle in degrees
```

```
alpha = atand(UydUx)
```

```
alpha =
9.0574
```
