# Supplementary material for: Objects with three orthogonal symmetry planes: Oblique driving forces and Stokes flow motion
Source: PLoS One. 2026 Jul 6;21(7):e0352508. doi: 10.1371/journal.pone.0352508 (PMC13336483; doi:10.1371/journal.pone.0352508)
Supplement: S4 File — PDF file containing the Matlab live script used in section 3.4.2. (PDF) [file pone.0352508.s004.pdf]

```
clearvars
theta = pi/4;
eta = 0.001; % dynamic viscosity 1.00 mPa-s
k_B = 1.38E-23; % Boltzmann's constant
T = 293.15; % Temperature 20 C
% minor axes of ellipsoid
a = 50E-9; % a = 50 nm, L1 = 100 nm
b = 5E-9; % b = 5 nm, L2 = 10 nm
c = 10E-9; % c = 10 nm, L3 = 20 nm

fa = @(x) 1./((a^2+x).*sqrt((a^2+x).*(b^2+x).*(c^2+x)));
qa = integral(fa, 0, inf)
```

```
qa =
2.6476e+22
```

```
fb = @(x) 1./((b^2+x).*sqrt((a^2+x).*(b^2+x).*(c^2+x)));
qb = integral(fb, 0, inf)
```

```
qb =
5.1888e+23
```

```
fc = @(x) 1./((c^2+x).*sqrt((a^2+x).*(b^2+x).*(c^2+x)));
qc = integral(fc, 0, inf)
```

```
qc =
2.5464e+23
```

```
zeta_1 = (16*pi*eta/3)*(b^2 + c^2)/(b^2*qb + c^2*qc)
```

```
zeta_1 =
5.4490e-26
```

```
zeta_2 = (16*pi*eta/3)*(a^2 + c^2)/(a^2*qa + c^2*qc)
```

```
zeta_2 =
4.7530e-25
```

```
zeta_3 = (16*pi*eta/3)*(a^2 + b^2)/(a^2*qa + b^2*qb)
```

```
zeta_3 =
5.3443e-25
```

```
D_r = k_B*T/zeta_1 % rotational diffusion coefficient
```

```
D_r =
7.4242e+04
```

```
tau = 0.5/D_r % rotational relaxation time about long axis
```

```
tau =
6.7347e-06
```

```
omega = 1/tau
```

```
omega =
1.4848e+05
```

```
freq = omega/(2*pi)
```

```
freq =
2.3632e+04
```
